# Online Variational Bayesian Filtering-Based Mobile Target Tracking in Wireless Sensor Networks

**DOI:** 10.3390/s141121281

**Published:** 2014-11-11

**Authors:** Bingpeng Zhou, Qingchun Chen, Tiffany Jing Li, Pei Xiao

**Affiliations:** 1 School of Information Science & Technology, Southwest Jiaotong University, Chengdu 610031, China; E-Mail: qcchen@swjtu.edu.cn; 2 Department of Electrical & Computer Engineering, Lehigh University, Bethlehem, PA 18015, USA; E-Mail: jingli@ece.lehigh.edu; 3 The Institute for Communication Systems, University of Surrey, Guildford, Surrey GU2 7XH, UK; E-Mail: p.xiao@surrey.ac.uk

**Keywords:** online tracking, Bayesian network, variational Bayesian filtering, WSN

## Abstract

The received signal strength (RSS)-based online tracking for a mobile node in wireless sensor networks (WSNs) is investigated in this paper. Firstly, a multi-layer dynamic Bayesian network (MDBN) is introduced to characterize the target mobility with either directional or undirected movement. In particular, it is proposed to employ the Wishart distribution to approximate the time-varying RSS measurement precision's randomness due to the target movement. It is shown that the proposed MDBN offers a more general analysis model via incorporating the underlying statistical information of both the target movement and observations, which can be utilized to improve the online tracking capability by exploiting the Bayesian statistics. Secondly, based on the MDBN model, a mean-field variational Bayesian filtering (VBF) algorithm is developed to realize the online tracking of a mobile target in the presence of nonlinear observations and time-varying RSS precision, wherein the traditional Bayesian filtering scheme cannot be directly employed. Thirdly, a joint optimization between the real-time velocity and its prior expectation is proposed to enable online velocity tracking in the proposed online tacking scheme. Finally, the associated Bayesian Cramer–Rao Lower Bound (BCRLB) analysis and numerical simulations are conducted. Our analysis unveils that, by exploiting the potential state information via the general MDBN model, the proposed VBF algorithm provides a promising solution to the online tracking of a mobile node in WSNs. In addition, it is shown that the final tracking accuracy linearly scales with its expectation when the RSS measurement precision is time-varying.

## 1. Introduction

Online tracking for a mobile node has attracted tremendous interests with the rapid advances in WSNs, which has also opened up many new tracking-based applications, such as smart shopping, smart homes, elderly people monitoring, intelligent transportation and military reconnaissance [1–4]. To fully meet the increasing demands of emerging applications, we need not only low-cost circuits, but also (more) efficient online tracking algorithms [5]. Existing online tracking algorithms for WSNs [6–11] can be roughly classified into two categories: range-free and range-based methods. Different types of mobile targets are considered in range-based/free online tracking, such as active and passive targets [12,13]. A customized classification procedure based on a support vector machine is proposed in [12] to investigate the passive tracking problem. In this paper, we focus on the active online tracking problem only. Among various (active) range-based schemes, there is an important subclass that uses the received signal strength (RSS) as the sole modality for localization and tracking [5,6,9,14,15]. The research interests in the RSS-based online tracking schemes can be attributed to two major factors: RSS measurements are available in all environments for almost every node that has a wireless communication function, no matter how small or cheap the node may be; and satisfactory localization and tracking accuracy can be achieved based on RSS measurements [14].

Nonetheless, technical obstacles still remain for the RSS-based online target tracking problem: firstly, how to generally characterize the random location transitions of a mobile target with either directional or undirected movement and how to alleviate the distortion caused by nonlinear observations, especially in a deep and random fading environment. Tackling these challenges requires the development of a new analysis model and new algorithms, which is the primary goal of this paper.

Diverse models have been developed to cope with the RSS-based online target tracking problem in WSNs. The general dynamic system model [16] and the random walking model [15,17] are two typical examples assumed in previous analysis. However, none of these previous models attempts to fully characterize the moving properties of the mobile target. For example, the general dynamic system model provides a general framework to capture the target's movement; however, it does not consider more underlying statistics of the system parameters, such as the a priori information of RSS precision. The random walking model can be utilized to characterize the target's movement via using an extended Gaussian model; however, it does not include the useful directional information of the moving target. Obviously, these kinds of prior information about the moving direction and other system parameters are important for online target tracking.

The online tracking for a mobile target can be solved under the Bayesian filtering (BF) framework [16,18]. However, the existence of nonlinear distortion and non-Gaussian noise in the observation would make the Bayesian prediction and updating analytically intractable. To circumvent this obstacle, a variety of modified or alternative approaches have been proposed in the literature, such as the extended Kalman filtering scheme in [19,20], the unscented Kalman filtering in [21], the Laplace approximation-based filtering (LAF) method in [11], the variational Bayesian filtering (VBF) algorithm in [8,22], the belief propagation scheme in [23] and various particle filtering algorithms in [7,15,16]. As the suboptimal implementation of BF, both the extended Kalman filtering and unscented Kalman filtering schemes seek to derive the analytical solutions via approximating the nonlinear observation functions in the original online target tracking problem with some parameterized methods. In the same way, the LAF algorithm employs a second-order approximation of the nonlinear function within the BF framework. Unfortunately, these parameterized algorithms are not robust enough to handle the nonlinear/non-Gaussian filtering problem. As a result, the achieved tracking accuracy is limited owing to the approximation errors [16,24]. In addition, the particle filtering algorithm offers an alternative solution to the online tracking problem and can be incorporated into any dynamic system model. In theory, the particle set can be employed to approximate an arbitrary probability distribution function if the number of particles is large enough; thus, it is capable of deriving a relatively accurate solution. Nonetheless, when the shape of the involved state transition distribution and that of the likelihood distribution become quite different from each other, the particle efficiency tends to deteriorate drastically [10,16]. Moreover, when the system model is characterized by a multi-layer Bayesian network (for example, the random walking model in [15,17]), the particle filtering algorithm will become too complicated to be implemented, since now, a variety of particle sets are needed to characterize the various variables involved. The online variational Bayesian inference (VBI) algorithm [22,25,26] and the belief propagation approach [23,27] represent another set of techniques to resolve the online target tracking problem. In fact, the belief propagation approach is equivalent to the BF framework, as illustrated in [23]. By replacing the real objective posterior pdf with another approximate pdf in terms of the minimum Kullback–Leibler divergence (KLD) in between both pdfs, the VBI-based filtering approaches can achieve a rather satisfactory approximation accuracy [25,26]. Moreover, the VBI algorithm can be realized in either a parameterized or a non-parameterized manner, which will be completely dependent on whether the involved functions or distributions can be analytically expressed or not. All previous research efforts suggest that, in order to derive an effective online target tracking algorithm, an efficient mathematical approach is highly desirable to deal with the nonlinear observation functions with non-Gaussian observation noises, in particular over shadowing and fading environments. Of course, the mobile target tracking problem can also be solved by using a forward-backward method in an offline manner, wherein all history and future data are available. In this paper, we just focus on online tracking, where only observations up to the current time instant are available, and the current target location is tracked in real time. Moreover, the online tracking is equivalent to a forward (and offline) tracking method, which jointly utilizes all observations (up to the current instant, as well), since in the online tracking framework, all history information at previous observations has been included by the *a posteriori* of the last state.

In this paper, online tracking for a mobile node is investigated, and the target location is identified in real time from observations just up to the current time instant. In order to fully characterize the movement of mobile target with either directional or non-directional movement and to incorporate as much as possible the related *a priori* information, a multi-layer dynamic Bayesian network (MDBN) is developed in this paper. This MDBN model offers a more general analysis model by incorporating the implicit prior statistical information of both the target movement and observation, which can be utilized to improve the online tracking capability by exploiting the Bayesian statistics. The proposed MDBN model can also subsume the random walking model as a special case. In the MDBN model, a velocity parameter is explicitly incorporated to characterize either the directional or non-directional movement of the mobile target. Since it is difficult to capture the initial moving velocity in advance, a joint optimization between the hidden velocity variable and its prior parameters is proposed to enable the online velocity tracking for the VBF algorithm. Based on the MDBN model, a mean-field VBF algorithm is developed to deduce the approximation to each posterior pdf. (It should be noted that the VBF algorithm is not the only algorithm that can be utilized to solve the online target tracking with directional and time-varying observation precision. In fact, based on the MDBN framework, other algorithms, such as particle filtering [16], can be employed to solve this kind of problem. However, there will be another problem that should be taken into account, such as the computational complexity of algorithms, as argued in Section 3.4.)

It is shown that the approximation to the objective individual pdf can be sufficiently characterized by the associated Markov blanket (MB). In addition, since the RSS measurement precision is time-varying when the target is moving, in order to reveal its effect on the achieved tracking performance, it is proposed to utilize a Wishart distribution to approximate its randomness. For the sake of a fair benchmark for the proposed VBF-based online target tracking scheme, a general Bayesian Cramer–Rao lower bound (BCRLB) is developed based on the MDBN model. Meanwhile, the specific BCRLB associated with the location tracking is also derived, which highlights the importance of the prior knowledge of the underlying moving direction in performing accurate tracking. Both the associated BCRLB analysis and the numerical simulation results are presented to validate that the proposed MDBN-based VBF algorithm provides a promising technique to realize online tracking of a mobile node in WSNs.

The remainder of this paper is organized as follows. Section 2 formulates the system model and the online target tracking problem in WSNs. The MDBN-based VBF online target tracking scheme is proposed in Section 3. The associated Bayesian CRLB for the MDBN-based VBF algorithm is derived in Section 4. Simulation results are presented in Section 5 to assess the mobile target tracking performance in different scenarios. Finally, Section 6 concludes the paper.

## 2. System Model

In this section, a multi-layer dynamic Bayesian network (MDBN) will be introduced to characterize the target mobility and the observation randomness, based on which, the VBF algorithm will be derived in the next section.

### 2.1. Nodes Deployment

Consider a WSN consisting of a mobile target and *M* anchor sensors, which are assumed to be randomly distributed within the deployed area. The locations of all anchor nodes are assumed to be known beforehand in which the *i* — th anchor's location is denoted by a D-dimensional column vector s*^i^*. Let S:= $\text{S}:={\left\{{\text{s}}^{i}\right\}}_{i=1}^{M}$ denote both the set of all sensors in the WSN and their location coordinates whenever no ambiguity arises. The location of the mobile target at time *t* is denoted by x*_t_*.

**Figure 1.**
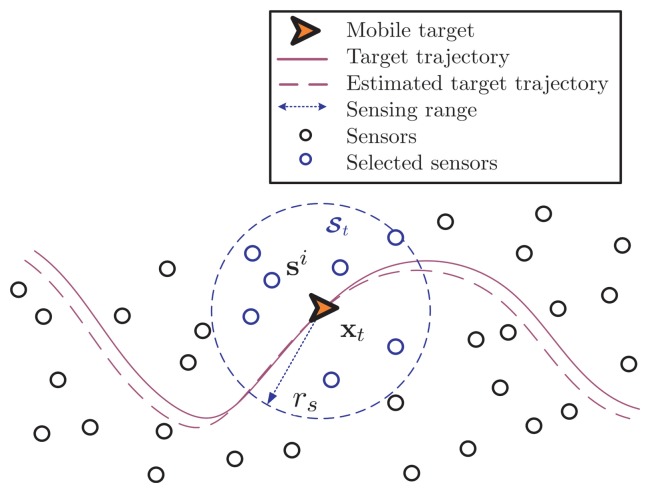
Illustration of the sensors deployment and the mobile target moving. Herein, the dotted circle denotes the sensing range within which the sensors can sense the target, and all of these nodes that perceive the target at time *t* inside the sensing circle form the cluster 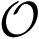
*_t_*.

As illustrated in Figure 1, the mobile target continues to communicate with its neighboring anchor nodes, so that the mobile target can obtain the RSS measurements relative to all anchor nodes within its vicinity. At time *t*, all of the sensors within the valid sensing range *r_s_* of the mobile target (the dotted circle in Figure 1) will formulate a reference cluster [9] to perform the online tracking for the mobile target. Define the reference cluster 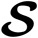
_*t*_ = {s*^i^ |* ║s*^i^* — x_*t*_║_2_ ≤ *r_s_*, ∀*i* = 1 : *M*} and assume *M_t_* is the total number of reference nodes in the cluster at time *t*, where ║·║_2_ represents the *ℓ*_2_-norm over an Euclidean space ℝ^D^ of dimension D. For convenience, the *i —* th reference node in the cluster 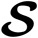
_*t*_ at time *t* is denoted by ${\text{s}}_{t}^{i}$ in the following analysis.

### 2.2. State Transition Model

In practice, the mobile target may travel inside the deployment area, either in a completely random manner or following a predetermined path, possibly with a time-varying velocity. Furthermore, in order to establish a general framework to characterize the target movement, the prior information should be, as much as possible, incorporated into the system. Hence, in this subsection, a new MDBN mobility model is introduced.

Assume the current (unknown) target position x*_t_* locates inside an area around a center (the current location expectation) *ν_t_, i.e.*,

(1) $${\text{x}}_{t}=\mu_{t}+{℘}_{t}$$

where the offset *℘_t_* is assumed to be a Gaussian distributed variable with zero mean and precision matrix **Λ***_t_*, namely, *℘_t_*∼ 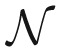 (*℘_t_*|0, **Λ***_t_*). Hence, the current target location follows x*_t_* ∼ 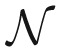
(**x***_t_*|(*μ_t_*,Λ*_t_*). In addition, we assume that the expectation *μ_t_* transits from its previous state *μ*_*t*−1_ plus the current velocity *ν_t_* with an offset *ς_t_*,

(2) $$\mu_{t}=\mu_{t-1}+\nu_{t}+\varsigma_{t}$$

where the offset *ς_t_* can be considered as the equivalent noise of this transition system, which is also assumed to be a Gaussian variable, *i.e., ς_t_* ∼ 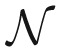
(*ς_t_*|0, U). Thus, we have *μ_t_∼* 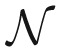
(*μ _t_ |μ*_*t*-1_ + *ν_t_*, U). Moreover, the velocity *ν_t_* at each time instant is assumed to be time-varying with a Gaussian distribution with a prior mean *υ* and a precision matrix V, namely *ν_t_ ∼* 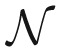
(*ν_t_|υ*,V). It should be noted that the above position transition model can subsume the completely random walking model as a special case when the associated velocity *ν_t_* = 0. Now, we go back to the target location transition model in Equation (1) and consider the precision matrix Λ*_t_* of location offset *℘_t_*. The mobile target with either directional or undirected movement may travel with a time-varying precision Λ*_t_*. Thus, in the sense of generality, we introduce a Wishart hyperpriorfor Λ*_t_*, which is the conjugate prior of the precision of a Gaussian distributed variable. Hence,

(3) $$\Lambda_{t}∼\text{w}\left(\Lambda_{t}|\sum ,\beta \right),$$

where ∑ stands for the corresponding prior scale matrix, and the scalar *β* is the associated degree of freedom (DOF). This model can subsume the case wherein the location transition precision is a fixed constant (for example, when the associated Wishart expectation ∑*β* goes to infinity).

Based on the above formulation, the corresponding state transition model (*i.e.*, the MDBN mobility model) can be summarized as below:

(4) $$\{\begin{array}{ccc} {\text{x}}_{t} & ∼ & \text{N}\left(\varepsilon_{t},\Lambda_{t}\right)\\ \mu_{t} & ∼ & \text{N}\left(\mu_{t}|\mu_{t-1}+\nu_{t},\text{U}\right)\\ \nu_{t} & ∼ & \text{N}\left(\nu_{t}|\upsilon ,\text{V}\right)\\ \Lambda_{t} & ∼ & \text{W}\left(\Lambda_{t}|\sum ,\beta \right) \end{array}$$

where the target location transition is formulated as an extended Gaussian model [22,28,29], *i.e.*, its expectation *μ_t_* and the precision matrix Λ*_t_* are also assumed to be random variables. We can see that the mobile target transition can be characterized by the evolution of its mean variable *μ_t_*. Here, all of the involved vectors and matrices are defined in the ℝ^D^ space and symmetric matrix space 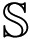
^D×D^ respectively.

### 2.3. Measurement Model

There are three popular measurement approaches in sensing the geometric information between the target and the reference nodes: the relative distance, the relative angle and the connectivity. As addressed before, we focus on the RSS-based tracking scheme, but the ideas and the methods developed herein can be readily extended to other modality measurements. The sensor nodes that have perceived the target will reply to the target, and the target can thus measure the RSS as below [14]:

(5) $$z_{t}^{i}=h^{i}\left({\text{x}}_{t}\right)+\in_{t}^{i},$$

where the scalar ${\text{z}}_{t}^{i}$ represents the RSS (in dB scale) related to the *i —* th reference node, and the scalar $\in_{t}^{i}$ represents the Gaussian noise with zero-mean and precision parameter w*_t_, i.e.*, $\in_{t}^{i}∼\text{N}(\in_{t}^{i}|0,{\text{w}}_{t})$. Here, *h^i^*(x*_t_*) is the measuring function, depending on the relative position of the target with respect to sensor ${\text{s}}_{t}^{i}$, which is defined as [14]:

(6) $$h^{i}\left({\text{x}}_{t}\right)=\phi -10\gamma \log_{10}{\left\|{\text{s}}_{t}^{i}-{\text{x}}_{t}\right\|}_{2},$$

where *ϕ = P_t_ — L*_0_ + 10*γ*log_10_ *d*_0_, *L*_0_ represents the power loss corresponding to the reference distance *d*_0_, which can be determined in the process of system calibration. *γ* is the path loss exponent within the range of [2, 4] [30], and *P_t_* is the transmit power of all nodes. In practical online target tracking applications, the RSS measurement precision *w_t_* is possibly time-varying when the target is moving. To capture these effects, a hyperprior pdf is introduced to model *w_t_* with a Wishart distribution, which is the conjugate prior of the precision of a Gaussian distributed variable. Hence,

(7) $${\text{w}}_{t}\sim \text{W}\left({\text{w}}_{t}|\text{W},\psi \right),$$

where the positive scalar W stands for its prior scale and *ψ* represents the associated DOF. In the following, all observations from all reference nodes in 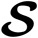
*_t_* are stacked in a *M_t_*-dimensional column vector, *i.e.*, $z_{t}={\left[z_{t}^{1},\cdots ,z_{t}^{M_{t}}\right]}^{\text{T}}$.

### 2.4. Problem Formulation

Before formulating the online target tracking problem, we introduce several essential definitions as follows, which help to explicate the fundamental concepts in the MDBN model.

#### Definition 1

Complete variable *α_t_*: The complete variable *α_t_* is defined as a variable set, which consists of all individual variables in the MDBN model, *i.e., α_t_* := {x*_t_, μ_t_, v_t_*, Λ*_t_, w_t_*}, *e.g.*, the target position x*_t_*, the RSS measurement precision w*_t_* and the involved hidden variables {*μ_t_,v_t_*,Λ*_t_*}. At the same time, let $\Theta_{t}:=\left\{\text{N}|\alpha_{t}^{\text{N}}\in \alpha_{t}\right\}$ stand for the associated index set of all individual variables.

#### Definition 2

Markov random variable: *Among all individual variables* $\alpha_{t}^{n}$ *in α_t_, the variable whose current state* $\alpha_{t}^{n}$ *only depends on its previous state* $\alpha_{t-1}^{n}$ *is defined as the Markov random variable*. In this specific MDBN model, only *μ_t_* depends on its previous state *μ*_*t*−1_, while the other states are independent of their parent states. Hence, the specific Markov random variable is *μ_t_*.

#### Definition 3

**Figure 2.**
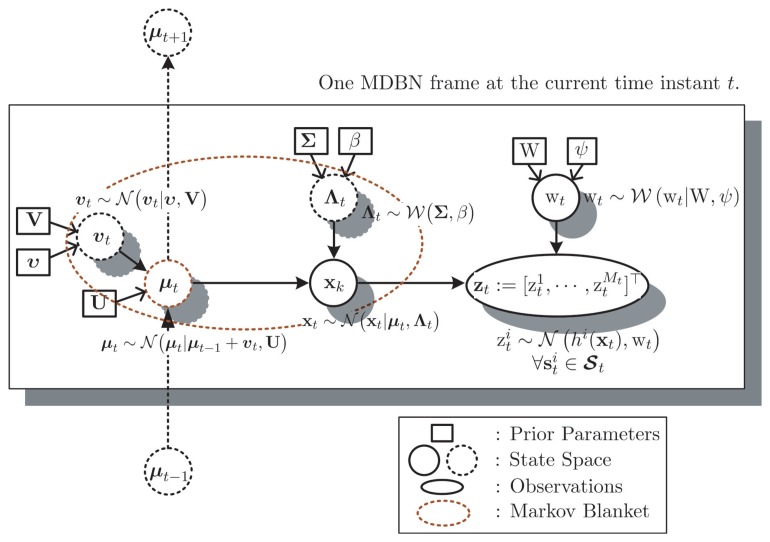
A general multi-layer dynamic Bayesian network (MDBN) model for the online tracking problem. This graph presents a temporal Bayesian network at the current time instant *t*. Here, the dotted circles denote the hidden variables, which cannot be directly observed, while the solid circle denotes the variables that can be measured. These variables together formulate the state space of MDBN.

Markov blanket: The Markov blanket (MB) of a variable $\alpha_{t}^{n}$ in a Bayesian network within one time instant is defined as a set of variables, which consists of the variable's parents, children and its parents' other children, which is denoted as $\text{B}(\alpha_{t}^{\text{N}})$ [31]. A specific example of the MB 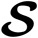 is depicted in Figure 2, where the variables inside the dotted ellipse formulate the MB of *μ_t_*, *i.e.*, 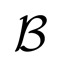: *=* {x*_t_*,Λ*_t_*,*ν_t_*}.

A general MDBN model characterizing the online target tracking problem, which is transformed from the state transition model Equation (4) together with the observation model Equations (5) and (7), can be configured as illustrated in Figure 2. As shown, the assumed MDBN contains three layers. The first layer is the so-called prior parameters layer, which contains some dependent parameters of the assumed prior distributions, such as *υ*, U, ∑, W, and so on. The second layer is the state space layer which consists of complete variables *α_t_*, *i.e.*, {x_t_, *μ_t_*, Λ*_t_*, *ν_t_*, w*_t_*}. The third layer is the observation layer, which contains the observations data z*_t_*.

Now, the online tracking problem for the mobile node is defined as follows.

#### Definition 4

Online target tracking: Given reference node locations ${\left\{{\text{s}}_{t}^{i}\right\}}_{\forall i=1:M_{t}}$ together with their measurement sequences z_1:_*_t_* up to current time instant *t*, how can we deduce the real-time target position?

## 3. The VBF-Based Online Target Tracking Scheme

### 3.1. Variational Bayesian Inference

Since it is difficult to directly find the exact closed-form expression for *p*(*α*_t_|z_1:_*_t_*) due to the existence of nonlinear observation functions in WSNs, we turn to find an alternative pdf *q*(*α*_t_) to approximate the objective posterior pdf *p*(*α*_t_|z_1:_*_t_*) via minimizing the Kullback-Leibler divergence (KLD) between them. The utilized KLD metric is defined as [26]:

(8) $$D_{\text{KL}}\left[q||p\right]=\int q\left(\alpha_{t}\right)\ln\left(\frac{q\left(\alpha_{t}\right)}{p\left(\alpha_{t}|z_{1:t}\right)}\right)\text{d}\alpha_{t},$$

in which the approximate pdf is assumed to be factorized as follows

(9) $$q\left(\alpha_{t}\right)=\prod_{\forall n\in \Theta_{t}}q\left(\alpha_{t}^{n}\right)=q\left({\text{x}}_{t}\right)q\left(\nu_{t}\right)q\left(\nu_{t}\right)q\left(\Lambda_{t}\right)q\left({\text{w}}_{t}\right),$$

where $q(\alpha_{t}^{n})$ stands for the approximation to the individual posterior pdf $p(\alpha_{t}^{i}|{\text{z}}_{1:t})$. In addition, we assume that the approximate posterior pdfs are independent of each other. A mean-field VBF can be utilized to design a distributed Bayesian tracking scheme, where each approximate distribution $q(\alpha_{t}^{i})$ that approximates the individual posterior pdf $p(\alpha_{t}^{i}|z_{1:t})$ can be derived as [26]:

(10) $$q\left(\alpha_{t}^{i}\right)\propto \exp{\langle \ln p\left(z_{1:t},\alpha_{t}\right)\rangle }_{\Pi_{j\neq i}q\left(\alpha_{t}^{j}\right)},$$

where ${\langle f(\alpha )\rangle }_{q(\alpha )}=\int f(\alpha )q(\alpha )\text{d}\alpha .$ and ∝ denotes that the left is proportional to the right. In Equation (10), the approximate distribution $q(\alpha_{t}^{i})$ is formulated with all of its complementary variables ${\left\{\alpha_{t}^{i}\right\}}_{\forall_{j\neq i}}:={\tilde{\alpha }}_{t}^{i}$. However, within the MDBN model, not all complementary variables $\left\{{\tilde{\alpha }}_{t}^{i}\right\}$ contribute to the derivation of $q(\alpha_{t}^{i})$. To approximate $q(\alpha_{t}^{i})$ more concisely, we have (see Appendix A):

(11) $$q\left(\alpha_{t}^{i}\right)=\exp{\langle \ln p\left(\alpha_{t}^{i}\beta \left(\alpha_{t}^{i}\right)\right)\rangle }_{q\left(\beta \left(\alpha_{t}^{i}\right)\right)},\left(11\right)$$

where $\text{B}(\alpha_{t}^{i})$ denotes the MB of variable $\alpha_{t}^{i}$ in the proposed MDBN model. This indicates that, if a variable $\alpha_{t}^{j}$ is not in the MB $\text{B}\left(\alpha_{t}^{i}\right)$ of variable $\alpha_{t}^{i}$, the terms associated with $\alpha_{t}^{j}$ have nothing to do with $\alpha_{t}^{j}$, and thus, they can be regarded as a multiplicative constant for $q(\alpha_{i}^{t})$. As a result, the approximate distribution $q(\alpha_{t}^{i})$ can be sufficiently characterized by the pdfs with respect to its MB $\text{B}(\alpha_{t}^{i})$. In the same way, all of the approximate distributions in the online target tracking problem can be given by:

(12) $$\begin{array}{c} q\left({\text{x}}_{t}\right)\propto \exp{\langle \ln p\left({\text{x}}_{t},\text{B}\left({\text{x}}_{t}\right)\right)\rangle }_{q\left(\text{B}\left({\text{x}}_{t}\right)\right)},\\ q\left(\mu_{t}\right)\propto \exp{\langle \ln p\left(\mu_{t},\text{B}\left(\mu_{t}\right)\right)\rangle }_{q\left(\text{B}\left(\mu_{t}\right)\right)},\\ q\left(\Lambda_{t}\right)\propto \exp{\langle \ln p\left(\Lambda_{t},\text{B}\left({\text{x}}_{t}\right)\right)\rangle }_{q\left(\text{B}\left(\Lambda_{t}\right)\right)},\\ q\left(\nu_{t}\right)\propto \exp{\langle \ln p\left(\nu_{t},\text{B}\left(\nu_{t}\right)\right)\rangle }_{q\left(\text{B}\left(\nu_{t}\right)\right)},\\ q\left({\text{w}}_{t}\right)\propto \exp{\langle \ln p\left({\text{w}}_{t},\text{B}\left({\text{w}}_{t}\right)\right)\rangle }_{q\left(\text{B}\left({\text{w}}_{t}\right)\right)}, \end{array}$$

where the involved MBs are given by:

(13) $$\begin{array}{l} \beta \left({\text{x}}_{t}\right)=\left\{z_{t},\mu_{t},\Lambda_{t},{\text{w}}_{t}\right\},\\ \beta \left(\mu_{t}\right)=\left\{{\text{x}}_{t},\nu_{t},\Lambda_{t}\right\},\\ \beta \left(\Lambda_{t}\right)=\left\{{\text{x}}_{t},\mu_{t}\right\},\\ \beta \left(\nu_{t}\right)=\left\{\mu_{t}\right\},\\ \beta \left({\text{w}}_{t}\right)=\left\{z_{t},{\text{x}}_{t}\right\}. \end{array}$$

### 3.2. Variational Bayesian Filtering

Before developing the MDBN-based VBF algorithm, we aim at revealing the underlying knowledge of the mobile target to provide Bayesian prediction, which can be further incorporated into the VBF-based online target tracking scheme.

#### 3.2.1. State Transition pdf

According to the considered MDBN model in Equation (4), the state transition distribution can be given by:

(14) $$\begin{array}{ll} p\left(\alpha_{t}|\alpha_{t-1}\right) & =p\left({\text{x}}_{t}|\mu_{t},\Lambda_{t}\right)p\left(\mu_{t}|\mu_{t-1}+\nu_{t}\right)p\left(\nu_{t}\right)p\left(\Lambda_{t}\right)p\left({\text{w}}_{t}\right)\\ & =\text{N}\left({\text{x}}_{t}|\mu_{t},\Lambda_{t}\right)\text{N}\left(\mu_{t}|\mu_{t-1}+\nu_{t},\text{U}\right)\text{N}\left(\nu_{t}|\upsilon ,V\right)\text{W}\left(\Lambda_{t}|\sum ,\beta \right)\text{W}\left({\text{w}}_{t}|\text{W},\phi \right)\\ & =p\left({\text{x}}_{t},\mu_{t},\nu_{t},\Lambda_{t}{\text{w}}_{t}|\mu_{t}-1\right). \end{array}$$

we can see that only *μ_t_* relates to its previous state *μ_t-_*_1_ (as defined in Definition 2) and the approximate distribution *q*(*α_t_*) can be sequentially updated only based on the transition pdf with respect to the Markov variables *μ_t_* and *μ_t-_*_1_. In fact, this conclusion has already been utilized in the derivation of formula Equation (A.1) (see Appendix A).

#### 3.2.2. Approximate Bayesian Prediction

Based on the state transition distribution, the state prediction pdf can be derived. Now, suppose each approximate pdf $q(\alpha_{t-1}^{i})$ has been obtained, and assume it is Gaussian distributed; then, according to Bayes's rules, the state prediction distribution *p*(*α_t_*|z_1:_*_t_*_-1_) can be approximated as:

(15) $$\begin{array}{ll} p\left(\alpha_{t}|z_{1:t-1}\right) & =\int p\left(\alpha_{t}|\alpha_{t-1}\right)p\left(\alpha_{t-1}|z_{1:t-1}\right)\text{d}\alpha_{t-1}\approx \int p\left(\alpha_{t}|\alpha_{t-1}\right)q\left(\alpha_{t-1}\right)\text{d}\alpha_{t-1}\\ & =\int p\left({\text{x}}_{t}|\mu_{t},\Lambda_{t}\right)p\left(\mu_{t}|\mu_{t-1}+\nu_{t},\text{U}\right)p\left(\nu_{t}\right)p\left(\Lambda_{t}|\sum ,\beta \right)p\left({\text{w}}_{t}\right)q\left(\alpha_{t-1}\right)\text{d}\alpha_{t-1}\\ & =p\left({\text{x}}_{t}|\mu_{t},\Lambda_{t}\right)p\left(\nu_{t}\right)p\left(\Lambda_{t}|\sum ,\beta \right)p\left({\text{w}}_{t}\right)\int p\left(\mu_{t}|\mu_{t-1}+\nu_{t},\text{U}\right)q\left(\mu_{t-1}\right)\text{d}\mu_{t-1}\\ & =p\left({\text{x}}_{t}|\mu_{t},\Lambda_{t}\right)p\left(\nu_{t}|\upsilon ,V\right)p\left(\Lambda_{t}|\sum ,\beta \right)p\left({\text{w}}_{t}|\text{W},\phi \right)q_{p}\left(\mu_{t}|\nu_{t},\text{U}\right). \end{array}$$

It can be observed that the approximate state prediction distribution can be sequentially updated, only based on the approximation distribution *q*(***μ****_t_*_−1_) and its transition function. Herein, the approximate prediction pdf of the mean variable is defined as:

(16) $$q_{p}\left(\mu_{t}|\upsilon_{t},\text{U}\right)=\int p\left(\mu_{t}|\mu_{t-1}+\upsilon_{t},\text{U}\right)q\left(\mu_{t-1}\right)\;d\mu_{t-1}.$$

Since the mean-transition pdf *p*(***μ****_t_*|***μ****_t_*_−1_+***ν****_t_*, **U**) and the approximate posterior pdf *q*(***μ****_t_*_-1_) are both assumed to be Gaussian distributed, $q(\mu_{t-1})=\text{N}(\mu_{t-1}|\mu_{t-1}^{*},{\text{U}}_{t-1}^{*})$, hence the approximate prediction pdf of the mean variable can also be assumed to be Gaussian distributed, *i.e.*,

(17) $$q_{p}\left(\mu_{t}|\upsilon_{t},\text{U}\right)=N\left(\mu_{t}|\mu_{t}^{p},{\text{U}}_{t}^{p}\right),$$

where the prediction expectation $\mu_{t}^{p}=\mu_{t-1}^{*}+\upsilon_{t}$ and the associated prediction precision ${\text{U}}_{t}^{p}={\left({\left({\text{U}}_{t-1}^{*}\right)}^{-1}+{\text{U}}^{-1}\right)}^{-1}$. Assume the prior pdf *p*(***υ**_t_*) of the velocity variable is also Gaussian distributed; the marginalized approximate prediction pdf of the mean variable can be derived as:

(18) $$\begin{array}{ll} q_{p}^{\upsilon }\left(\mu_{t}\right) & =\int q_{p}\left(\mu_{t}|\upsilon_{t},\text{U}\right)p\left(\upsilon_{t}|\upsilon ,\text{V}\right)d\upsilon_{t}\\ & =\int \text{N}\left(\mu_{t}|\mu_{t}^{p},{\text{U}}_{t}^{p}\right)\text{N}\left(\upsilon_{t}|\upsilon ,\text{V}\right)d\upsilon_{t}\\ & ≔\text{N}\left(\mu_{t}|\mu_{t}^{\upsilon },{\text{U}}_{t}^{\upsilon }\right), \end{array}$$

where the marginalized expectation $\mu_{t}^{\upsilon }$ can be obtained as $\mu_{t}^{\upsilon }=\mu_{t-1}^{*}+\upsilon$ and the associated precision is obtained as ${\text{U}}_{t}^{\upsilon }={\left({\left({\text{U}}_{t-1}^{*}\right)}^{-1}+{\text{U}}^{-1}+{\text{V}}^{-1}\right)}^{-1}$. According to the Gaussian distribution properties, the extended Gaussian pdf whose dependent parameter is also Gaussian distributed can be marginalized as a Gaussian pdf, as well.

#### 3.2.3. Likelihood pdf

Recalling the RSS observation model in formula Equation (5), the RSS observation $z_{t}^{i}$ from the reference sensor ${\text{s}}_{t}^{i}$ in the cluster 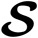
*_t_* is given by $z_{t}^{i}=h^{i}({\text{x}}_{t})+\epsilon_{t}^{i}$, where the measurement noise is denoted as $\epsilon_{t}^{i}∼N(0,{\text{w}}_{t})$ with a time-varying precision w*_t_* ∼ 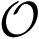 (w*_t_*|W, *ψ*). Assume the observation $z_{t}^{j}$ from **s***^i^* is independent of any other observation $z_{t}^{j}$, ∀*j* ≠ *i*; then, the corresponding joint likelihood pdf can be given by:

(19) $$L\left({\text{z}}_{t}|{\text{α}}_{t}\right)=\prod_{i=1}^{M_{t}}\frac{{\left|w_{t}\right|}^{\frac{1}{2}}}{\sqrt{2\pi }}\exp\left(-\frac{1}{2}w_{t}\;{\left(z_{t}^{i}-h^{i}\left(x_{t}\right)\right)}^{2}\right),$$

where *M_t_* is the number of all available reference nodes in the current cluster 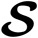
*_t_*.

#### 3.2.4. Approximate *A Posteriori* Update

At each VBI iteration of a BF step, when deducing the posterior approximation $q(\alpha_{t}^{i})$, it is assumed that all of the other approximations $q(\alpha_{t}^{j})$ ∀*j* ≠ *i*, have been determined. In addition, we assume the prior distribution $p(\alpha_{t}^{j})⫫p(\alpha_{t}^{i})$, ∀ ≠*i*, where • ⫫ • means the left side is independent of the right side. Based on VBI theory (see Equation (12)) and the above analysis associated with BF, the variational approximate posterior $q(\alpha_{t}^{i})$ for each individual variable $\left(\alpha_{t}^{i}\right)$ can be derived as:

(20) $$\begin{array}{l} q\left(x_{t}\right)\propto N\left(x_{t}|\langle \mu_{t}\rangle ,\langle \Lambda_{t}\rangle \right)\cdot \overset{M_{t}}{\underset{i=1}{\text{∏}}}N\left(z_{t}^{i}|h^{i}\left(x_{t}\right),\langle w_{t}\rangle \right),\\ q\left(\mu_{t}\right)\propto N\left(\mu_{t}|\mu_{t}^{*},{\text{U}}_{t}^{*}\right),\\ q\left(\Lambda_{t}\right)\propto W\left(\Lambda_{t}|\sum_{t}^{*},\beta_{t}^{*}\right),\\ q\left(\upsilon_{t}\right)\propto N\left(\upsilon_{t}|\upsilon_{t}^{*},{\text{V}}_{t}^{*}\right),\\ q\left(w_{t}\right)\propto W\left(w_{t}|W_{t}^{*},\psi_{t}^{*}\right), \end{array}$$

where all individual approximate posterior parameters are given by:

(21) $$\begin{array}{ll} \mu_{t}^{*} & ={\left({\text{U}}_{t}^{*}\right)}^{-1}({\text{U}}_{t}^{p}{\langle \mu_{t}^{p}\rangle }_{\nu_{t}}+\langle \Lambda_{t}\rangle \langle x_{t}\rangle ),\\ {\text{U}}_{t}^{*} & ={\text{U}}_{t}^{p}+\langle \Lambda_{t}\rangle ;\\ \sum_{t}^{*} & ={\left(\sum^{-1}+{\text{X}}_{t}\right)}^{-1},\\ \beta^{*} & =\beta +1,\\ \nu_{t}^{*} & ={\left({\text{V}}_{t}^{*}\right)}^{-1}\left({\text{U}}_{t}^{p}{\langle \nu_{t}^{\#}\rangle }_{\mu_{t}}+\text{V}\nu \right);\\ {\text{V}}_{t}^{*} & ={\text{U}}_{t}^{p}+\text{V},\\ \psi_{t}^{*} & =\psi +M_{t},\\ {\text{W}}_{t}^{*} & ={\left(\overset{M_{t}}{\underset{i=1}{\text{∑}}}{\left({\text{W}}_{t}^{i}\right)}^{-1}+{\text{W}}^{-1}\right)}^{-1}. \end{array}$$

Additionally, the involved expectations are:

(22) $$\begin{array}{ll} {\langle \mu_{t}^{p}\rangle }_{\nu_{t}} & =\mu_{t-1}^{*}+\langle \nu_{t}\rangle ,\\ {\text{X}}_{t} & :={\langle ({\text{x}}_{t}-\mu_{t}){\left({\text{x}}_{t}-\mu_{t}\right)}^{⊤}\rangle }_{{\text{X}}_{t},\mu_{t}}\\ & =\langle {\text{x}}_{t}{\text{x}}_{t}^{⊤}\rangle -\langle {\text{x}}_{t}\rangle \langle \mu_{t}^{⊤}\rangle -\langle \mu_{t}\rangle \langle {\text{x}}_{t}^{\text{⊤}}\rangle +\langle \mu_{t}\mu_{t}^{⊤}\rangle ,\\ \langle \mu_{t}\mu_{t}^{⊤}\rangle & ={\left({\text{U}}_{t}^{*}\right)}^{-1}+\langle \mu_{t}\rangle \langle \mu_{t}^{⊤}\rangle ,\\ {\langle \nu_{t}^{\#}\rangle }_{\mu_{t}} & =\langle \mu_{t}\rangle -\mu_{t-1}^{*},\\ {\text{W}}_{t}^{*} & :={\langle {\left(z_{t}^{i}-h^{i}({\text{x}}_{t})\right)}^{2}\rangle }_{{\text{x}}_{t}}^{-1};\\ \langle \mu_{t}\rangle & =\mu_{t}^{*},\langle \Lambda_{t}\rangle =\beta^{*}\sum_{t}^{*},\\ \langle {\text{w}}_{t}\rangle & =\psi_{t}^{*}{\text{W}}_{t}^{*},\langle v_{t}\rangle =v_{t}^{*}. \end{array}$$

where ()^⊤^ denotes the matrix transpose, the operator ${\langle •\rangle }_{\alpha_{t}^{i}}$ is equivalent to ${\langle •\rangle }_{q(\alpha_{t}^{i})}$ and the expectation ${\langle \alpha_{t}^{i}\rangle }_{\alpha_{t}^{i}}$ with respect to its own approximate posterior pdf is simplified as $\langle \alpha_{t}^{i}\rangle$. The detailed derivations can be found in Appendix B.

We can observe from Equation (20) that the approximate posterior *q*(x*_t_*) is the product of a Gaussian pdf and some irregular pdfs, which cannot be expressed in a closed form solution due to the log-normal pdfs in RSS observations. Thus, we resort to a particle-based approximation to *q*(x*_t_*) by using an importance sampling method, wherein the particles are given by ${\left\{\chi_{t}^{\left(m\right)},\omega_{t}^{\left(m\right)}\right\}}_{m=1}^{N_{s}}$ where $\left\{\chi_{t}^{\left(m\right)}\right\}∼N({\text{x}}_{t}|\langle \mu_{t}\rangle ,\langle \Lambda_{t}\rangle )$ and the weight $\omega_{t}^{\left(m\right)}\propto \prod_{i=1}^{M_{t}}N\left({\text{z}}_{t}^{i}|h^{i}\left(\chi_{t}^{\left(m\right)}\right),\langle {\text{w}}_{t}\rangle \right)$. Hence, the corresponding expectations associated with x*_t_* can be approximated as:

(23) $$\langle {\text{x}}_{t}\rangle \approx \sum_{\forall m}\omega_{t}^{\left(m\right)}\chi_{t}^{\left(m\right)},\langle {\text{x}}_{t}{\text{x}}_{t}^{⊤}\rangle \approx \sum_{\forall m}\omega_{t}^{\left(m\right)}\chi_{t}^{\left(m\right)}\chi_{t}^{(m)⊤}.$$

except for x*_t_*, the rest of the approximate posterior pdfs $q(\alpha_{t}^{i})$ ( $\forall \alpha_{t}^{i}\in \alpha_{t}$ and $\alpha_{t}^{i}\neq {\text{x}}_{t}$) can be derived in a closed form based on the proposed VBF algorithm. Given particle set ${\left\{\chi_{t}^{\left(m\right)},\omega_{t}^{\left(m\right)}\right\}}_{m=1}^{N_{s}}$ to characterize the approximate posterior *q*(**x***_t_*), the scalar ${\text{W}}_{t}^{i}$ in Equation (21) can be reformulated as:

(24) $${\left({\text{W}}_{t}^{i}\right)}^{-1}={\left({\text{z}}_{t}^{i}\right)}^{2}-2{\text{z}}_{t}^{i}{\langle h^{i}\left({\text{x}}_{t}\right)\rangle }_{{\text{x}}_{t}}+{\langle {\left(h^{i}\left({\text{x}}_{t}\right)\right)}^{2}\rangle }_{{\text{x}}_{t}}.$$

Additionally, the involved expectations with respect to function *h^i^* (x*_t_*) can be approximated by:

(25) $$\begin{array}{ll} {\langle h^{i}({\text{x}}_{t})\rangle }_{{\text{X}}_{t}} & \approx \overset{N_{s}}{\underset{m=1}{\text{∑}}}\omega_{t}^{\left(m\right)}h^{i}(\chi_{t}^{\left(m\right)}),\\ {\langle {\left(h^{i}({\text{x}}_{t})\right)}^{2}\rangle }_{{\text{X}}_{t}} & \approx \overset{N_{s}}{\underset{m=1}{\text{∑}}}\omega_{t}^{\left(m\right)}{\left(h^{i}(\chi_{t}^{\left(m\right)})\right)}^{2}. \end{array}$$

Hence, all scale parameters ${\left\{{\text{W}}_{t}^{i}\right\}}_{\forall i=1:M_{t}}$ involved in Equation (22) can be accordingly approximated based on Equations (24) and (25) by using the particle set ${\left\{\chi_{t}^{\left(m\right)},\omega_{t}^{\left(m\right)}\right\}}_{m=1}^{N_{s}}$.

#### 3.2.5. Joint Optimization for Online Velocity Tracking

In practical online target tracking applications, it is probably difficult to know the real value of prior parameter *ν* of offset *ν_t_* in advance. Hence, in order to provide an online velocity tracking for the VBF,a joint optimization for the prior velocity expectation *ν* is proposed to provide an adaptive tracking for the target velocity *ν_t_*. The optimal *ν̂* can be deduced via minimizing the corresponding mean square errors, *i.e.*,

(26) $$\hat{\nu }=\arg\underset{\nu^{\prime }}{\min}\int {\left(\nu^{\prime }-v\right)}^{⊤}\left(\nu^{\prime }-\nu \right)p(\nu |z_{1:t})\text{d}\nu .$$

This problem is similar to the estimation of complete variable *α_t_*, which also attempts to find the minimum mean squared error (MMSE) estimation and needs to deduce the posterior *p*(*ν*|**z**_1:_*_t_*) at first. Due to there being no closed-form expression for *p*(*ν*|**z**_1:_*_t_*), here, we still resort to the VBI method to find the approximate density *q*(*ν*) to approximate the real posterior *p*(*ν*|**z**_1:_*_t_*).

From the defined MDBN model shown in Figure 2, we know that the corresponding MB 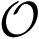
(**ν**) = ***ν**_t_*. Based on Equation (12), the desired approximate posterior pdf relative to **ν** can be formulated as:

(27) $$\begin{array}{ll} q(v) & =\exp{\langle \text{In }p\left(v,B(v)\right)\rangle }_{q\left(B(v)\right)}\\ & =\exp{\langle \text{In }p(v,v_{t})\rangle }_{q(v_{t})}\\ & =\exp{\langle \text{In}\left(N(v_{t}|v,\text{V)}p(v)\right)\rangle }_{v_{t}}\\ & \propto p(v)\cdot N(v|{\langle v_{t}\rangle }_{v_{t}},\text{V})\\ & \propto N(v|{\langle v_{t}\rangle }_{v_{t}},\text{V}), \end{array}$$

wherein *p*(*ν*) is regarded as a uniform distribution, since there is no prior information about the prior velocity mean. Based on the MMSE-related optimization in Equation (26), the corresponding optimal estimation is formulated as the posterior expectation, *i.e.*, *ν̂* ∫ **ν***p*(*ν*|**z**_1:_*_t_*) d*ν*. Hence, the associated approximate estimation is the corresponding approximate posterior expectation, *i.e.*, *ν̂* = 〈*ν_t_*〉 *_ν_t__*. At the same time, the expectation $\nu_{t}^{*}$ of the approximate posterior pdf *q* (*ν_t_*) in Equation (21) (wherein the real-time velocity *ν_t_* is also estimated as the approximate posterior expectation based on the VBF algorithm) should be modified in each VBF iteration as:

(28) $$\nu_{t}^{*}={\left({\text{V}}_{t}^{*}\right)}^{-1}\left({\text{U}}_{t}^{p}{\langle \nu_{t}^{\#}\rangle }_{\mu_{t}}+\text{V}\hat{\nu }\right).$$

#### 3.3. The VBF Scheme Realization

It is assumed that the locations of the reference anchors 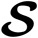 will be delivered to the mobile target through the request-reply procedure. Once all reference anchor locations are received at the target, the proposed VBF scheme can iteratively identify the approximation to the posterior pdf *p*(**x***_t_*|**z**_1:_***t***) from **z***_t_*, given the *a priori* knowledge of some initial parameters, including the path loss component γ *L*_0_, during the system calibration stage. Assume that the target is equipped with sufficient hardware and software to afford the computation complexity of the VBF algorithm. Hence, the optimal estimation **x̂***_t_* can be derived as its posterior expectation 〈**x***_t_*〉. The pseudo-code description of the proposed VBF scheme is presented in Algorithm 1.



**Algorithm 1:** VBF-Online Target Tracking Algorithm
**Input**
$\mu_{t-1}^{*}$, 
$\Lambda_{t-1}^{*}$, 
${\text{z}}_{t}^{\text{s}}$.1**Initialization**: 
$\mu_{0}^{*}$, 
$\Lambda_{0}^{*}$, **U**, V, Σ,*β*, W, *ψ, v.*2**For**
*t* = 1 : *K*3 Detect the target, construct the references set 

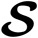
_*t*_ and gather current observations z*_t_*;4 Initialize 〈*v_t_*〉 = *v*, compute 
${\langle \mu_{T}^{P}\rangle }_{\upsilon_{t}}=\mu_{t-1}^{*}+\langle \upsilon_{t}\rangle$ and 
${\text{U}}_{t}^{p}={\left({\left({\text{U}}_{t-1}^{*}\right)}^{-1}+{\text{U}}^{-1}+{\text{V}}^{-1}\right)}^{-1}$;5 Initialize the temporal variational parameters, 
$\mu_{t}^{*}={\langle \mu_{t}^{p}\rangle }_{\upsilon_{t}}$, 
${\text{U}}_{t}^{*}=3{\text{U}}_{t}^{p}$, 
$\upsilon_{t}^{*}=\langle \upsilon_{t}\rangle$, 
${\text{V}}_{t}^{*}+\frac{1}{3}\text{V}$, β* = *β* + 1, 
$\Sigma_{t}^{*}={\left(3{\left(U_{t}^{p}\right)}^{-1}+\Sigma^{-1}\right)}^{-1}$, 
$\psi_{t}^{*}=\psi +M_{t}$, and 
${\text{W}}_{t}^{*}=\frac{1}{3}\text{W}$;6 Compute the expectations 
$\langle \mu_{t}\rangle =\mu_{t}^{*}$, 
$\langle \Lambda_{t}\rangle =\beta^{*}\Sigma_{t}^{*}$, 
$\langle \mu_{t}\mu_{t}^{Τ}\rangle ={\left({\text{U}}_{t}^{*}\right)}^{-1}+\langle \mu_{t}\rangle \langle \mu_{t}^{Τ}\rangle$
$\langle {\text{w}}_{t}\rangle =\psi_{t}^{*}{\text{W}}_{t}^{*}$ and 
${\langle \upsilon_{t}^{+}\rangle }_{\mu_{t}}=\langle \mu_{t}\rangle -\mu_{t-1}^{*}$;7 **While**
*not converge*
**do**8  Generate *N_s_* particles 
${\left\{\chi_{t}^{\left(m\right)},\omega_{t}^{\left(m\right)}\right\}}_{m=1}^{N_{s}}$ to approximate to *q*(x*_t_*), *i.e.*,
$\chi_{t}^{\left(m\right)}∼N\left(\langle \mu_{t}\rangle ,\langle \Lambda_{t}\rangle \right)$, 
$\omega_{t}^{\left(m\right)}\propto \prod_{i=1:M_{t}}p\left({\text{z}}_{t}^{i}|h^{i}\left({\text{x}}_{t}\right),\langle {\text{w}}_{t}\rangle \right)$;9  Calculate the associated expectation and correlation under *q*(x*_t_*), 
$\langle {\text{x}}_{t}\rangle \approx \sum_{\forall m}\omega_{t}^{\left(m\right)}\chi_{t}^{\left(m\right)}$, 
$\langle {\text{x}}_{t}{\text{x}}_{t}^{Τ}\rangle \approx \sum_{\forall m}\omega_{t}^{\left(m\right)}\chi_{t}^{\left(m\right)}\chi_{t}^{\left(m\right)Τ}$;**Vm**10  **For** i=1: *M_t_*11   
${\langle h^{i}\left({\text{x}}_{t}\right)\rangle }_{{\text{x}}_{t}}\approx \sum_{\forall m}\omega_{t}^{\left(m\right)}h^{i}\left(\chi_{t}^{\left(m\right)}\right),{\langle {\left(h^{i}\left({\text{x}}_{t}\right)\right)}^{2}\rangle }_{{\text{x}}_{t}}\approx \sum_{\forall m}\omega_{t}^{\left(m\right)}{\left(h^{i}\left(\chi_{t}^{\left(m\right)}\right)\right)}^{2}$;12  **End**13  Update each variational posterior parameter as follows, 
${\text{U}}_{t}^{*}=\langle \Lambda_{t}\rangle +{\text{U}}_{t}^{p}$,
$\mu_{t}^{*}={\left({\text{U}}_{t}^{*}\right)}^{-1}\left({\text{U}}_{t}^{p}{\langle \mu_{t}^{p}\rangle }_{\upsilon_{t}}+\langle \Lambda_{t}\rangle \langle {\text{x}}_{t}\rangle \right)$, 
$\upsilon_{t}^{*}={\left({\text{V}}_{t}^{*}\right)}^{-1}\left({\text{U}}_{t}^{p}{\langle \upsilon_{t}^{\#}\rangle }_{\mu_{t}}+\text{V}\upsilon \right)$, 
${\text{V}}_{t}^{*}={\text{U}}_{t}^{p}+\text{V}$,
${\text{X}}_{t}:=\langle {\text{x}}_{t}{\text{x}}_{t}^{Τ}\rangle -\langle {\text{x}}_{t}\rangle \langle \mu_{t}^{Τ}\rangle -\langle \mu_{t}\rangle \langle {\text{x}}_{t}^{Τ}\rangle +\langle \mu_{t}\mu_{t}^{Τ}\rangle$, 
$\Sigma_{t}^{*}={\left(\Sigma^{-1}+{\text{X}}_{t}\right)}^{-1}$,14  **For**
*i* = 1 : *M_t_*15   
${\left({\text{W}}_{t}^{i}\right)}^{-1}={\left({\text{z}}_{t}^{i}\right)}^{2}-2{\text{z}}_{t}^{i}{\langle h^{i}\left({\text{x}}_{t}\right)\rangle }_{{\text{x}}_{t}}+{\langle {\left(h^{i}\left({\text{x}}_{t}\right)\right)}^{2}\rangle }_{{\text{x}}_{t}}$,16  **End**17  
$W_{t}^{*}={\left(\sum_{i=1}^{M_{t}}{\left({\text{W}}_{t}^{i}\right)}^{-1}+{\text{W}}^{-1}\right)}^{-1}$;18  Update the associated expectations 
$\langle \mu_{t}\rangle =\mu_{t}^{*}$, 
$\langle \Lambda_{t}\rangle =\beta^{*}\Sigma_{t}^{*}$, 
$\langle {\text{w}}_{t}\rangle =\psi_{t}^{*}{\text{W}}_{t}^{*}$, 
$\langle \upsilon_{t}\rangle =\upsilon_{t}^{*}$, 
${\langle \upsilon_{t}^{\#}\rangle }_{\mu_{t}}=\langle \mu_{t}\rangle -\mu_{t-1}^{*}$
$\langle \mu ,\mu_{t}^{Τ}\rangle ={\left({\text{U}}_{t}^{*}\right)}^{-1}+\langle \mu_{t}\rangle \langle \mu_{t}^{Τ}\rangle$ and
${\langle \mu_{t}^{p}\rangle }_{\upsilon_{t}}=\mu_{t-1}^{*}+\langle \upsilon_{t}\rangle$,19 **End**20 Estimate the target location, ^x*_t_* = 〈x*_t_*〉;21**End****Output:** ^x*_t_*
$\mu_{t}^{*}$, 
$\Lambda_{t}^{*}$


#### 3.4. Algorithm Complexity

The computational complexity of the proposed VBF algorithm scales with 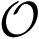
*(M_t_N_s_C_VBF_* 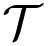), where *M_t_, N_s_* denote the current reference cluster size and the particle set size, respectively. 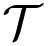 denotes the maximum iterations, such that the VBI iteration (see s = Steps 7-19 in Algorithm 1) in the VBF algorithm converges. Experimentally, 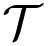 = 6 is generally sufficient for the VBI convergence. *C*_VBF_ is defined as the number of individual variables in the complete variable set *αt* of the VBF algorithm: *C*_VBF_ := Card{*α_t_*}. In the proposed VBF-based online target tracking scheme in this paper, *C*_VBF_ = 5. For a comparison, we consider a traditional particle filtering (PF) algorithm [16], where the transition distribution is a Gaussian transition model, and its proposed function is the transition distribution itself. The computational complexity of the PF algorithm scales with 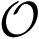
(*M_t_N_s_C*_PF_), if we assume that there is no time-varying observation precision in the PF algorithm, *i.e.*, the individual variables are only x_t_ and *v_t_*. In such a case, *C*_PF_ = 2; thus, the VBF is more complicated than the PF algorithm. (Nevertheless, it should be noted that, here, 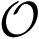
(*M_t_N_s_C*_PF_) and 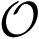 (*M_t_N_s_C*_VBF_ 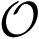) just reflect how the computation cost scales with the factors involved in the algorithms, rather than the exact amount of computation. To give a computationally fair comparison, we should specify how much computational cost is totally required to guarantee an equivalent tracking accuracy for different algorithms.) On the other hand, if the time-varying observation precision w*_t_* is considered in the PF algorithm, the PF's computational complexity will scale with $O\left(M_{t}N_{s}^{2}\right)$. Additionally, experimentally, *N_s_* = 200. In such a case, the PF algorithm will becomes more complicated than the proposed VBF algorithm. Moreover, if more system variables are considered in the PF algorithm, such as the random precision matrix μ*_t_* of the location offset ℘*_t_*, then the PF's computation cost will scale with $O\left(M_{t}N_{s}^{3}\right)$. Overall, as more underlying hyperprior statistics are considered, the computational cost will grow exponentially, as discussed in the PF-related introduction (see Section 1). Hence, the VBF algorithm is preferable to the PF algorithm when considering the randomness of RSS observation precision in the online target tracking problem.

### 4. Cramer-Rao Lower Bound

CRLB provides a general lower bound for any unbiased estimator, which is mathematically formulated as the inverse of the associated Fisher information matrix (FIM) [17]. In this section, a Bayesian CRLB for the VBF-based online location tracking is developed to provide a benchmark for the proposed VBF-based online target tracking scheme. Additionally, then, the CRLB for the MLE-based location tracking and the asymptotic analysis are derived for comparison purposes.

#### 4.1. Bayesian CRLB for VBF-Based Location Tracking

According to the VBF algorithm, the location tracking is designed based on its approximate posterior pdf *q*(x*_t_*). Hence, the VBF-based location tracking can be equivalently considered as an estimator based on *p*(x_t_|z_1:t_).

The Bayesian CRLB with respect to x_t_, denoted by $B_{\text{BF},t}^{\text{x}}$, is formulated as [17]:

(29) $$\begin{array}{l} B_{\text{BF},t}^{\text{x}}={\left(\underset{\text{BF based FIM}}{\underset{︸}{-{\text{E}}_{{\text{z}}_{t},{\text{x}}_{t}}\left\{\nabla_{{\text{x}}_{t},{\text{x}}_{t}^{⊤}}\ln\;p({\text{x}}_{t}\{{\text{z}}_{1:t})\right\}}}\right)}^{†}\\ ={\left(\underset{\text{Observation Knowledge}}{\underset{︸}{-{\text{E}}_{{\text{z}}_{t},{\text{x}}_{t}}\left\{\nabla_{{\text{x}}_{t},{\text{x}}_{t}^{⊤}}\ln\;p({\text{z}}_{t}|{\text{x}}_{t})\right\}}}\underset{\text{Prior Knowledge}}{\underset{︸}{-{\text{E}}_{{\text{z}}_{t},{\text{x}}_{t}}\left|\nabla_{{\text{x}}_{t},{\text{x}}_{t}^{⊤}}\ln\;p({\text{x}}_{t})\right\}}}\right)}^{†}\\ ={\left(J_{\text{MLE},t}^{\text{x}}+J_{\text{P},t}^{\text{x}}\right)}^{†}:={\left(J_{\text{BF},t}^{\text{x}}\right)}^{†}, \end{array}$$

where the symbol $\nabla_{{\text{x}}_{t},{\text{x}}_{t}^{Τ}}$ (•) denotes a second order derivative operator with respect to x*_t_*, 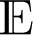
_z*t*,x*t*_ {•} denotes the expectation with respect to the distribution *p*(z*_t_*, x*_t_*) and † represents the pseudo-inverse. As shown in Equation (29), $B_{\text{BF},t}^{\text{x}}≜\left(J_{\text{MLE},t}^{\text{x}}+J_{\text{P},t}^{\text{x}}\right)$, (in other words, the BF framework, such as VBF, integrates the information from both observations and state prediction) where $J_{\text{MLE},t}^{\text{x}}$ stands for the MLE-based FIM (*i.e.*, the observation information), $J_{\text{P},t}^{\text{x}}$ stands for the state prediction-related FIM and $J_{\text{BF},t}^{\text{x}}$ stands for the BF-related CRLB at time *t*, with respect to the variable x*_t_*. Given the marginalized likelihood pdf in Equation (19), the MLE-based FIM $J_{\text{MLE},t}^{\text{x}}$ can be further formulated as:

(30) $$\begin{array}{ll} J_{\text{MLE},t}^{\text{X}} & =-{\text{E}}_{{\text{z}}_{t},{\text{x}}_{t}}\left\{\nabla_{{\text{x}}_{t},{\text{x}}_{t}^{\text{T}}}\ln p({\text{z}}_{t}|{\text{x}}_{t})\right\}=--{\text{E}}_{{\text{z}}_{t},{\text{x}}_{t}}\left\{\nabla_{{\text{x}}_{t},{\text{x}}_{t}^{\text{T}}}In\left(\overset{M_{t}}{\underset{i=1}{\text{∏}}}\int p({\text{z}}_{t}^{i}|{\text{x}}_{t})p({\text{w}}_{t})\text{d}w_{t}\right)\right\}\\ & =-\overset{M_{t}}{\underset{i=1}{\text{∑}}}{\text{E}}_{{\text{z}}_{t},{\text{x}}_{t}}\left\{\nabla_{{\text{x}}_{t},{\text{x}}_{t}^{\text{T}}}\ln\int p({\text{z}}_{t}^{i}|{\text{x}}_{t})p({\text{w}}_{t})\text{d}w_{t}\right\}\\ & \leq -\overset{M_{t}}{\underset{i=1}{\text{∑}}}{\text{E}}_{{\text{z}}_{t},{\text{x}}_{t}}\left\{\nabla_{{\text{x}}_{t},{\text{x}}_{t}^{\text{T}}}\int p({\text{w}}_{t})\text{In}p({\text{z}}_{t}^{i}|{\text{x}}_{t},{\text{w}}_{t})\text{d}w_{t}\right\}\\ & =\underset{{\text{R}}_{\text{MLE},t}^{\text{X}}}{\underset{︸}{-\overset{M_{t}}{\underset{i=1}{\text{∑}}}{\text{E}}_{{\text{z}}_{t},{\text{x}}_{t}}\left\{\nabla_{{\text{x}}_{t},{\text{x}}_{t}^{\text{T}}}\text{In}N({\text{z}}_{t}^{i}|h^{i}({\text{x}}_{t}),{\text{w}}_{t})\right\}.}} \end{array}$$

Herein, a Jensen's inequality about the logarithm function is utilized. One can see that the MLE-based FIM $J_{\text{MLE},t}^{\text{x}}$ is upper bounded by another FIM $R_{\text{MLE},t}^{\text{x}}$ given by (see Appendix C):

(31) $$R_{MLE,t}^{x}={\left(\frac{10\gamma }{\ln10}\right)}^{2}W\psi A_{t},$$

where the matrix **A***_t_* is:

(32) $$A_{t}=\sum_{i=1}^{M_{t}}\frac{\left(x_{t}-s_{t}^{i}\right){\left(x_{t}-s_{t}^{i}\right)}^{T}}{{\left\|x_{t}-s_{t}^{i}\right\|}_{2}^{4}}.$$

In fact, the matrix **A***_t_* represents the relative geometric information between the target and its reference nodes, while ${\left(\frac{10\gamma }{\ln10}\right)}^{2}$ W*ψ* stands for the environment information for the target tracking.

The state prediction-based FIM $R_{\text{P},t}^{\text{x}}$ can be similarly derived as:

(33) $$\underset{R_{P,t}^{x}}{\underset{︸}{\begin{array}{l} \;J_{P,t}^{x}=E_{z_{t},x_{t}}\left\{\nabla_{x_{t},x_{t}^{T}}\ln p\left(x_{t}\right)\right\}\\ =-E_{z_{t}},x_{t}\left\{\nabla_{xt,x_{t}^{T}}\ln\iint p\left(x_{t}|\mu_{t},\Lambda_{t}\right)q_{p}^{\upsilon }\left(\mu_{t}\right)p\left(\Lambda_{t}\right)d\mu_{t}d\Lambda_{t}\right\}\\ =-E_{z_{t}},x_{t}\left\{\nabla x_{t,x_{t}^{T}}\ln\int N\left(x_{t}|\mu_{t}^{\#},\Lambda_{t}^{\#}\right)W\left(\Lambda_{t}|\Sigma ,\beta \right)d\Lambda_{t}\right\}\\ \leq -E_{z_{t}},x_{t}\left\{\int \nabla x_{t,x_{t}^{T}}\ln\int N\left(x_{t}|\mu_{t}^{\#},\Lambda_{t}^{\#}\right)W\left(\Lambda_{t}|\Sigma ,\beta \right)d\Lambda_{t}\right\} \end{array}}}$$

where $\mu_{t}^{\#}=\mu_{t}+\mu_{t}^{\upsilon }$ and $\Lambda_{t}^{\#}={\left(\Lambda_{t}^{-1}+{\left({\text{U}}_{t}^{\upsilon }\right)}^{-1}\right)}^{-1}={\left(\Lambda_{t}^{-1}+{\left({\text{U}}_{t-1}^{*}\right)}^{-1}+{\text{U}}^{-1}+{\text{V}}^{-1}\right)}^{-1}$.Additionally:

(34) $$\underset{{\tilde{R}}_{P,t}^{x}}{\underset{︸}{\begin{array}{l} R_{P,t}^{x}=-E_{z_{t},x_{t},\Lambda_{t}}\left\{\nabla_{x_{t},x_{t}^{T}}\ln N\left(x_{t}|\mu_{t}^{\#},\Lambda_{t}^{\#}\right)\right\}\\ =-E_{\Lambda_{t}}\left\{\Lambda_{t}^{\#}\right\}=E_{\Lambda_{t}}\left\{{\left(U_{t-1}^{*}\right)}^{-1}+U^{-1}+\vee^{-1}+\Lambda_{t}^{-1}\right\}\\ \leq {\left({\left(U_{t-1}^{*}\right)}^{-1}+U^{-1}+\vee^{-1}{\left(E_{\Lambda_{t}}\left\{\Lambda_{t}\right\}\right)}^{-1}\right)}^{-1}\\ ={\left({\left(U_{t-1}^{*}\right)}^{-1}+U^{-1}+\vee^{-1}\beta -1\Sigma^{-1}\right)}^{-1}. \end{array}}}$$

Their relative order among these three bounds is as follows:

(35) $$J_{P,t}^{x}\leq R_{P,t}^{x}\leq {\tilde{R}}_{P,t}^{x}.$$

Thus, the state prediction-based FIM $J_{\text{P},t}^{\text{x}}$ is also upper bounded by ${\tilde{R}}_{\text{P},t}^{\text{x}}$. Furthermore, we have:

(36) $$\begin{array}{c} B_{BF,t}^{x}={\left(J_{MLE,t}^{x}+J_{P,t}^{x}\right)}^{†}\geq \underset{G_{BF,t}^{x}}{\underset{︸}{{\left(R_{MLE,t}^{x}+{\tilde{R}}_{P,t}^{x}\right)}^{†}}}\\ ={\left({\left(\frac{10\gamma }{\ln10}\right)}^{2}W\psi A_{t}+{\left(U_{t-1}^{*}\right)}^{-1}+U^{-1}+V^{-1}+{\left(\beta \Sigma \right)}^{-1}\right)}^{†}. \end{array}$$

We can find that only $R_{\text{MLE},t}^{\text{x}}$ relates to the environment information ${\left(\frac{10\gamma }{\ln10}\right)}^{2}$ in $B_{\text{BF},t}^{\text{x}}$ and $G_{\text{BF},t}^{\text{x}}$. If we just consider the time-varying property of the RSS measurement precision, the final VBF error bound $B_{\text{BF},t}^{\text{x}}$ only depends on the its expectation and has nothing to do with the other statistical characteristics, no matter how the precision varies with time. More specifically, the final VBF accuracy (which is defined as the inverse of the error) scales linearly with the expectation *ψ*W of the random precision.

Considering a long-term online tracking system (*t* = 1 : *K*), and suppose two scenarios where:

(a) the RSS measurement precision *w_t_* varies with time, *i.e., w_t_ = w1*,⋯, w*_K_*;
(b) the RSS measurement precision *wt* is invariant and equals ¯w*_t_*;

if ${\bar{w}}_{t}=\underset{K\to \infty }{\lim}\sum_{t=1}^{K}w_{t}/K$, then $B_{BF,t}^{x}\left(a\right)=B_{BF,t}^{x}\left(b\right)$ in which $B_{\text{BF},t}^{\text{x}}(a)$ and $B_{\text{BF},t}^{\text{x}}(b)$ stand for the CRLB corresponding with Scenarios (a) and (b), respectively.

This invariant phenomenon indicates to us that, in the practical online target tracking problem, even though the real RSS precision changes with time, when performing the VBF-based online tracking, we can equivalently consider the precision being fixed at its expectation value, such that the VBF algorithm can still achieve an equivalent tracking accuracy. Hence, if the expectation of the real time-varying precision is known in advance, the VBF-based online target tracking scheme can be performed with a lower computational complexity without reducing the tracking accuracy, where the time-varying precision is replaced by its expectation (a constant).

We know that the VBF-based online target tracking scheme is a specific realization of the BF; hence, the covariance of the VBF can be lower bounded as:

(37) $$\mathrm{cov}\left(x_{t}\right)\geq B_{BF,t}^{x}\geq G_{BF,t}^{x}.$$

That is to say, $G_{\text{BF},t}^{\text{x}}$ also serves as a (loose) lower bound of the location tracking errors. Let *e_t_* = ‖x̂_*t*_ − x_*t*_‖_2_ stand for the tracking error at time ***t***; the corresponding root mean squared error (RMSE) of the VBF-based online target tracking scheme is given by [14]:

(38) $$\sqrt{E\left\{e_{t}^{2}\right\}}=\sqrt{E\left\{{\left\|{\hat{x}}_{t}-x_{t}\right\|}_{2}^{2}\right\}}\geq \sqrt{tr\left(G_{BF,t}^{x}\right),}$$

where tr(•) is the trace of a square matrix.

According to Jensen's inequality, we know that the gap between the two sides of the associated inequality (e.g., Equations (30), Equations (33) and Equations (34)) tends to approach zero when the precision of the involved pdf goes to infinity. Hence, if all precisions of the involved pdf go to infinity, the new CRLB $G_{\text{BF},t}^{\text{x}}$ will approach $B_{\text{BF},t}^{\text{x}}$, *i.e.*,

(39) $$\underset{\gamma_{w,}\gamma_{\Lambda }\to \infty }{\lim}G_{BF,t}^{x}=B_{BF,t}^{x},$$

where **ϒ_w_** and **ϒa** denote the precision matrix of the Wishart distributed random variables **w***_t_* and **Λ***_t_*, respectively

#### 4.2. The CRLB for the MLE-Based Location Tracking

On the other hand, the CRLB of the MLE-based location tracking is formulated as:

(40) $$B_{MLE,t}^{x}={\left(J_{MLE,t}^{x}\right)}^{†}\geq \underset{G_{MLE,t}^{x}}{\underset{︸}{{\left(R_{MLE,t}^{x}\right)}^{†}}}.$$

Since no state prediction information is utilized in the MLE-based location tracking scheme, the achieved performance will depend on the RSS measurement information only

#### 4.3. Asymptotic Analysis

The Bayesian CRLB serves as the lower bound for our VBF-based online target tracking scheme, which characterizes the contribution of the information from not only the RSS observations, but also the state prediction information. Additionally, the state prediction-based FIM $\text{FIM}{\overset{∼}{R}}_{\text{P},t}^{\text{x}}$ corresponds to the prior information in the BF framework.

It can be observed from Equation Equations (34) that, if all involved precision matrices **V, U** and the scale matrix Σ go to zero, the SPI-related FIM will approach zero, *i.e.*,

(41) $$\underset{\Sigma ,V,U\to 0}{\lim}{\tilde{R}}_{P,t}^{x}=0,$$

which leads to:

(42) $$\begin{array}{l} J_{P,t}^{x}=0,\\ G_{BF,t}^{x}\to G_{MLE,t}^{x},\\ B_{BF,t}^{x}\to B_{MLE,t}^{x}\left(as\;\Sigma ,V,U\to 0\right). \end{array}$$

In this case the location prediction does not provide any useful information, the mobile target may move to any place within the whole deployment region at the next time instant. In such a case, the BCRLB for the BF-based online tracking scheme will degenerate to that of the MLE-based online tracking scheme.

On the other hand, if the state precisions **V**, **U** and the scale matrix Σ go to infinity, which implies the state transition function *p*(α*_t_|* α*_t-1_*) tends to be a Dirac function, it can provide a completely accurate state prediction. In this case, the BCRLB becomes:

(43) $$\underset{\Sigma ,V,U\to \infty }{\lim}B_{BF,t}^{x}={\left(U_{t-1}^{*}+R_{MLE,t}^{x}\right)}^{†}.$$

In such a case, the tracking accuracy at time t will depend on the previous tracking precision **INLINE** and the precision **INLINE** associated with the current observations. The BCRB analysis clearly shows us that a good model that is able to characterize and integrate the useful state transition information will play an important role in improving the BF-based online target tracking approach.

As for the BF-based CRLB and the MLE-based CRLB, we have the following proposition.

**Proposition1.** The BF-based CRLB is definitely lower than the MLE-based CRLB, *i.e.*,

(44) $$G_{BF,t}^{x}\leq G_{MLE,t}^{x}.$$

**Proof.** Based on Equations Equations (36) and Equations (40), we know that $G_{BF,t}^{x}{\left(R_{MLE}^{x}+{\tilde{R}}_{P,t}^{x}\right)}^{†}$, while $G_{MLE,t}^{x}={\left(R_{MLE,t}^{x}\right)}^{†}$. Because ${\tilde{R}}_{P,t}^{x}\geq 0$ and ${\tilde{R}}_{MLE,t}^{x}\geq 0$, thus we have $G_{BF,t}^{x}\leq G_{MLE,t}^{x}$

### 5. Simulation Analysis

In this section, we present extensive simulation results (such as the VBF errors, CRLBs and convergence properties) to evaluate the performance of the proposed VBF scheme.

#### 5.1. Simulation Introduction

In the following simulations, a particle filtering (PF) algorithm is used as a benchmark scheme to compare with the VBF algorithm. In order to clearly demonstrate the gain in tracking performance by using the proposed MDBN model, the observation precision is assumed to be deterministic in the PF algorithm; the transition distribution is assumed to be a Gaussian transition model (rather than the MDBN model), and its proposal function is the transition distribution itself (of course, the PF method can also be incorporated into the proposed MDBN model to capture the directional information), as the classical PF algorithm is performed [16], while our MDBN-based VBF algorithm takes into account both the precision's randomness and the mobility directionality.

In the assessment demonstrated below, we will use the root mean squared error averaged over various time instants in many repeated runs as a figure of merit for the proposed VBF-based online tracking scheme, and the RMSE is calculated as follows:

(45) $$e_{VBF}\;or\;e_{PF}=\sqrt{\frac{1}{LK}\sum_{t=1}^{K}\sum_{l=1}^{L}{\left\|{\hat{x}}_{t}^{\left(l\right)}-x_{t}\right\|}_{2}^{2}},$$

where ${\hat{x}}_{t}^{\left(l\right)}$ denotes ^x*_t_* in the *l* — th simulation of the VBF or PF algorithm, and *L* = 10^4^ simulation runs are performed for each setting. Other metrics used in the assessment include the RMSE of the BF-based CRLB *(i.e.*, G_BF_) and that of the MLE-based CRLB *(i.e.*, G_MLE_), which are defined as follows:

(46) $$\begin{array}{c} G_{BF}=\sqrt{\frac{1}{LK}\sum_{t=1}^{K}\sum_{l=1}^{L}tr\left(G_{BF,t}^{x,\left(l\right)}\right)},\\ G_{MLE}=\sqrt{\frac{1}{LK}\sum_{t=1}^{K}\sum_{l=1}^{L}tr\left(G_{MLE,t}^{x,\left(l\right)}\right)}, \end{array}$$

where $G_{BF,t}^{x\left(l\right)}$ and $G_{MLE,t}^{x\left(l\right)}$ denote the BF-based CRLB and the MLE-based CRLB at time *t* in the *l* — th simulation, respectively.

**Figure 3.**
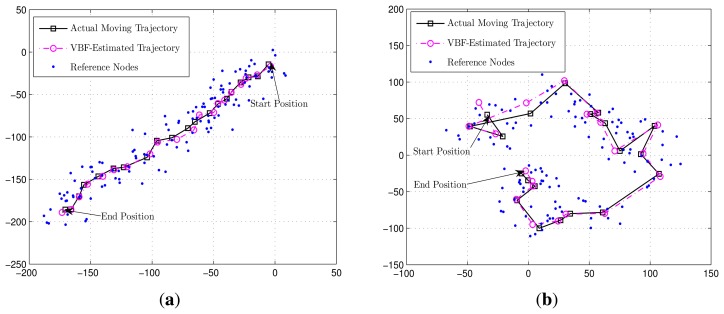
Illustrates of the variational Bayesian filtering (VBF) scheme for a mobile target. In both subfigures, the solid line with squares denotes the actual trajectory and the doted line with circles denotes the trajectory estimated by the VBF scheme. In both cases, some system parameters are set to be Σ = 1/5**I**, *β* = 10, W = 1/1500, *ψ* = 80, *M_t_* = 6. Other parameters are specified in the two subfigures, respectively. Note that the units in both x-axis and y-axis are meters. (**a**) The mobile target moves in a directional manner, where *υ* = 10**I** [m/s], **U** = 1/10**I** and **V** = 1/10**I**. In this case, the achieved RMSEs are: *e*_VBF_ = 3.24, G_MLE_ = 4.05 and G_BF_ = 3.24; (**b**) The mobile target moves without a directional trend, where ***υ*** = **0** [m/s], **U** = 1/900**I** and **V** = 1/10**I**. In this case, the achieved RMSEs are: *e*_VBF_ = 4.63, G_MLE_ = 4.59 and G_BF_ = 4.55.

In the simulations, all anchor sensors are deployed uniformly in the deployment area, and the mobile target is assumed to walk around with a time-varying velocity Two examples of the node deployment and mobile target trajectory are illustrated in Figure 3a,b, which show the directional and non-directional scenario, respectively The points (in blue) denote the ensemble of the reference nodes in various clusters *[* 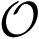
*]* at discrete time *t* = 1, 2, ⋯, *K (K* = 20 is shown in the plot). (The anchor nodes that do not provide service in this session are dropped from the plot.) Each cluster 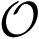 has a circular enclosure that is centered around the mobile target with a radius *r_s_* and usually includes *M_t_* reference nodes inside. At each time, the references in *St* are assumed to be uniformly distributed inside a circle area given by *Ct =* {˘x| ‖˘x — x*_t_*‖_2_ < *r*_s_}, in order to remove the influence of the references deployment.

A series of factors can affect the tracking performance, such as the variance of shadow fading, the variance of mobile target position shifting, the velocity of the mobile target, the number of reference nodes, and so on. In order to unveil the effect of these different factors on the VBF algorithm, we consider the simulation Scenarios B1-B4. The simulation setup of B5 is utilized to demonstrate the calculation convergen *γ* = 3, *P_T_* = 50, *L_0_* = 1, *d*_0_ = 1, *r_s_* = 20 [m] and *K* = 20 [s]. According to the computational complexity analysis in Section 3.4, we know that the complexity of the proposed VBF algorithm is in the order of 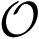
*(M_t_N_s_C*_VBF_ 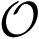
*)*, while the PF algorithm is 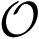 *(M_t_N_s_C*_PF_*).* In the context of the specific online tracking studied in this paper, we also know CV_BF_ = 5 and *C*_PF_ = 2. Hence, in order to give a computationally fair comparison between the PF and the VBF algorithm, we assume $N_{PF}=\frac{C_{VBT}T}{C_{PF}}NVBF$, where *N*_PF_ and *N*_VBF_ stand for the numbers of particles used in the following simulations with respect to the PF algorithm and the VBF algorithm, respectively. Experimentally, we set 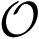 = 6 and *N*_VBF_ = 200; thus, *N*_PF_ = 3, 000 particles are employed in the PF algorithm in the following simulations.

#### 5.2. Numerical Results Analysis

##### 5.2.1. Influence of the Movement Directionality

The average velocity *ν* partly determines how regularly the mobile target moves. If *ν* is relatively large (or equivalently, the precisions V and U become relatively large), this means that the target moves with an apparent trend and that its movement is more regular. To assess the performance of the VBF algorithm over different velocity means, we consider Scenario B1 in this experiment, wherein *ν* varies in different cases. The corresponding simulations settings are given in Table 1.

**Figure 4.**
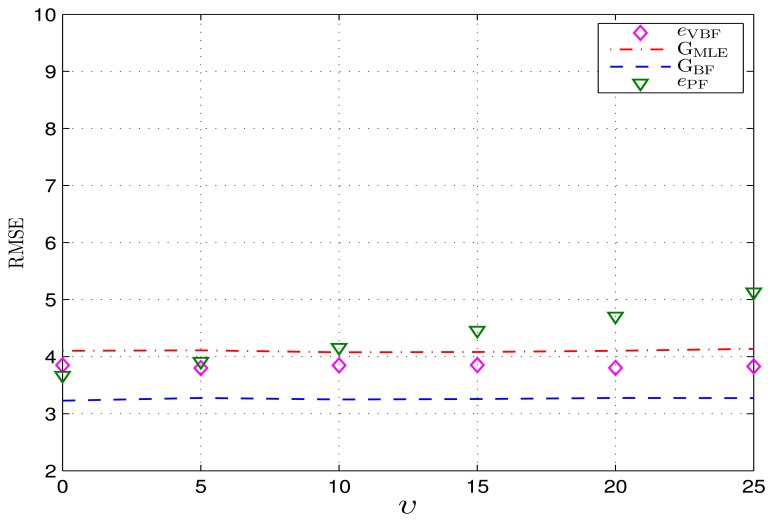
Achieved RMSEs over different movement velocity means. The y-axis is in meters.

The achieved RMSEs e_VBF_ of the proposed VBF scheme and two corresponding CRLBs G_BF_, G_MLE_ are shown in Figure 4. It is indicated that the final tracking error of the VBF algorithm does not depend on the velocity mean *ν*, since the associated MDBN model integrates a velocity variable, thus being capable of capturing the mobility information. While the traditional PF algorithm [16] just employs the general Gaussian transition model, which does not consider the target's movement directionality, thus, its final tracking performance depends on how directionally the target moves. In particular, if the target moves with a larger velocity, the particles employed in PF must have a larger distribution area to capture its movement; hence, the presentation accuracy of these particles is reduced, which results in a relatively poor tracking performance.

##### 5.2.2. Influence of the Movement Randomness

The location-transition precision U and the velocity-varying precision V jointly indicate how randomly or how directionally the mobile target moves. In order to assess the performance of the proposed VBF algorithm over different movement models, Scenario B2 is considered in this simulation, wherein U and V vary while other parameters are set to be fixed. The corresponding simulation settings are given in Table 1.

**Figure 5.**
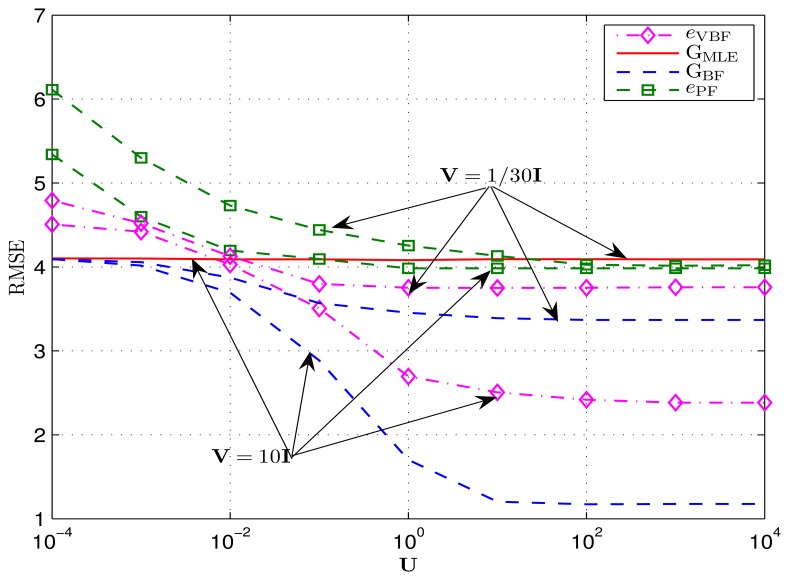
Achieved RMSEs by the VBF and particle filtering (PF) algorithm with different target mobilities. The *y*-axis is in meters.

The achieved RMSEs of the VBF algorithm e_VBF_ and the associated CRLBs G_BF_ and G_MLE_ are summarized in Table 2, wherein the top row values stand for $\sigma_{u}^{2}$, such that $U=\sigma_{u}^{-2}$, and the left column values stand for $\sigma_{u}^{2}$, such that $V=\sigma_{v}^{-2}$, respectively. As shown in the table, when both U and V are relatively large (which means the target moves rather regularly), the final RMSE of the proposed VBF algorithm is significantly lower than the MLE-based CRLB G_MLE_ (Note that, our emphasis is not to claim how wonderfully the VBF algorithm beats the MLE-based CRLB G_MLE_ here. We just attempt to provide an upper threshold for tracking errors of a Bayesian algorithm. If the resulting tracking error exceeds this threshold, the Bayesian tracking algorithm is bad. On the contrary, if the tracking error is lower than G_MLE_, then we say this algorithm is valuable, since the final tracking accuracy is not lower than the observation information, *i.e.*, the a priori statistics model in this Bayesian tracking algorithm works.) and gets close to the BF-related CRLB G_BF_. When U and V are very small, the VBF algorithm outperforms on par with G_MLE_, and the BF-based CRLB G_BF_ is only slightly better. Figure 5 shows the RMSEs achieved by both the VBF algorithm and the PF algorithm over different target mobilities, wherein U = 10^-4^I : 10^4^I while V = 1/30I : 10I. Refer to Table 1 (see B2) for other simulation settings. We can see that the VBF algorithm outperforms the PF algorithm, which follows from the fact that, the VPF algorithm exploits some potential state information for the online target tracking scheme via the general MDBN model.

##### 5.2.3. Influence of the Deep Shadow Fading

The scale parameter W and the associated DOF *ψ* jointly indicate how randomly the observation noise precision varies. In order to assess the performance of the proposed VBF-based online tracking algorithm over different levels of the observation noise distortion, Scenario B3 is considered in this experiment, wherein W and *ψ* change while U = 1=10I, V = 1=10I and other parameters are fixed, as shown in Table 1.

**Figure 6.**
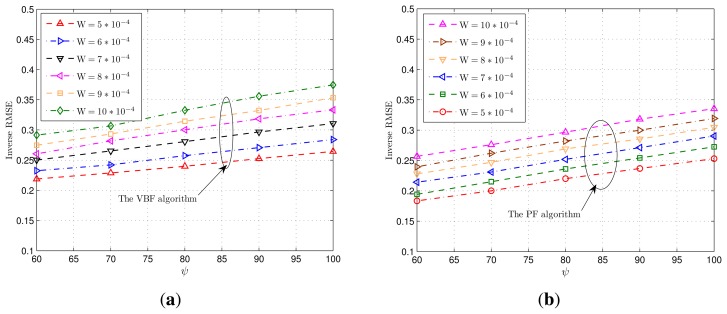
Tracking accuracy over different precision expectations. The *y*-axis is in meters. **(a)** Accuracies achieved by the VBF algorithm over different levels of deep shadow fading; **(b)** accuracies achieved by the PF algorithm over different levels of deep shadow fading.

The achieved RMSEs of the VBF scheme and associated positioning CRLBs, *i.e.*, e_VBF_, G_BF_ and G_MLE_, are summarized in Table 3, wherein the top row values stand for the DOF *ψ* and the left column values stand for the scale parameter W, respectively. As shown in the table, when W and *ψ* of the observation noise precision is very large, which means the noise distortion is not severe, the VBF scheme achieves lower tracking RMSE than the MLE-based CRLB G_MLE_, while it is slightly larger than the relaxed BF-related CRLB G_BF_. Since the BF-based CRLB G_BF_ is obtained by lower relaxation, it is reasonable that there is a gap between the achieved RMSE eVBF and G_BF_ In addition, when W and *ψ* of the observation noise precision get smaller, which means that the noise varies more wildly with large variance, the associated tracking error gets larger. In particular, the tracking accuracy can be defined as the inverse of the RMSE. The corresponding accuracies achieved by the VBF and the PF algorithms are presented in Figure 6a,b, respectively. It is shown that the achieved accuracies of either VBF or PF algorithm almost linearly scale with the expectation *Wψ*, as explicated in Equation Equations (36). Moreover, comparing Figure 6a with 6b one can see that the VBF algorithm outperforms the PF algorithm, since the VBF algorithm exploits some potential state information for online tracking via the general MDBN model.

**Figure 7.**
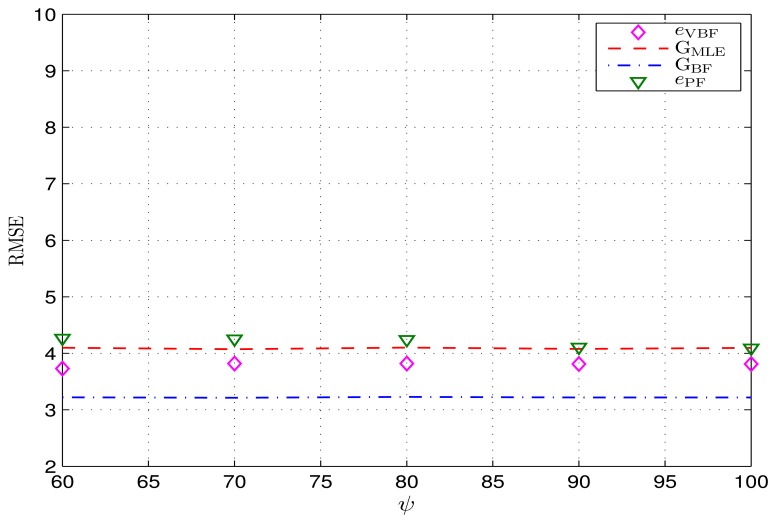
Achieved RMSEs over different deep shadow fading environments with the same expectation of w*_t_*. The *y*-axis is in meters.

In Figure 7, the achieved RMSEs of the VBF-based online tracking scheme and its CRLBs are simulated in different deep shadow fading environments, wherein the expectation is fixed, while theDOF *ψ* varies and the scaleWalso varies accordingly. As shown in Figure 7, the RMSEs eVBF achieved by using the VBF scheme almost remain unchanged when the expectations *Wψ*are set to be the same value 80=1500; so is the PF algorithm. Moreover, this invariant property also holds for two associated CRLBs G_BF_ and G_MLE_ in this case. Namely, the VBF scheme and its CRLB are just related to the expectation of the random noise precision only, with relevance to its variance or other related statistical characteristics. This phenomenon indicates that, if this expectation can be known beforehand, the VBF scheme (containing, but not limited to, the VBF) can achieve an equivalent tracking accuracy with lower computation cost when the time-varying precision is replaced by its expectation.

### 5.2.4. Influence of the References Cluster Size

**Figure 8.**
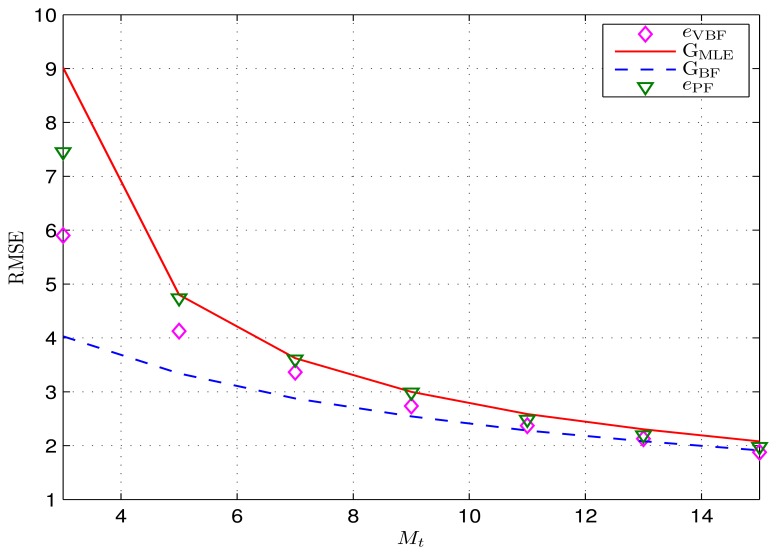
Achieved RMSEs with different reference cluster sizes. They *y*-axis is in meters.

At each time t, a temporal reference cluster


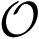


is formed around the mobile target, where the reference anchor sensors in the cluster provide location references. To evaluate the impact of the number of reference nodes, Figure 8 simulates Scenario B4, where the average size of the reference cluster increases from M_t_ = 3 to 15 (by increasing the density of the sensor node while keeping the sensing range r_s_ = 20). The other simulation settings are given in Table 1.

As expected, the tracking error of the proposed VBF scheme reduces with the increasing number of reference nodes. In addition, the proposed VBF algorithm outperforms the PF algorithm in terms of the tracking errors, since the general MDBN model exploits more *a priori* information for the VBF algorithm. We can also see that there still exits a gap between the VBF algorithm and the relaxed BF-based bound G_BF_, partly due to the relaxation in deriving *G*_BF_. Furthermore, the gap between *G*_BF_ and *G*_MLE_ reduces as the reference cluster size increases, since more reference nodes can provide more observation information for the target tracking. When there is a sufficient amount of observations, the Bayesian estimation will be equivalent to the MLE. Moreover, that indicates that six to nine reference nodes can achieve a reasonable positioning accuracy.

### 5.2.5. Convergence of the VBF Scheme

In order to examine the convergence properties of the proposed VBF scheme, *i.e.*, the convergence of the VBF algorithm for the target position estimation and convergence of the VBI algorithm for the position estimation, Scenario B5 is considered in this experiment, which consists of two cases. The corresponding simulations settings are given in Table 1.

The first case is to test the convergence of the position tracking errors in the VBF scheme. Practically, if the VBF scheme can accurately track the mobile target at the first time instant, then in the following time instants, the VBF scheme can still capture the mobile target trajectory, according to the classical BF theory. This result can be explicated by the fact that, in such a case, the posterior information provided by the first time instant is sufficiently accurate to predict the next state. On the other hand, if the initial values of prior parameters (such as U, V, W, *ψ*; especially the prior velocity mean *υ*) are not accurate, the achieved RMSE is likely to be very large, thus giving rise to a large initial tracking-error. (Note that, if the initializations of the movement precision U, V are slightly smaller than their true values, then the initial searching area of the VBF scheme can be larger, thus a better chance of capturing the moving target. Thus, there is smaller initial tracking error.) To test whether the proposed VBF scheme can quickly track the trajectory of mobile target in the presence of the initial positioning error, an initial error (equaling five) is factitiously introduced for the VBF scheme in this sub-experiment.

**Figure 9.**
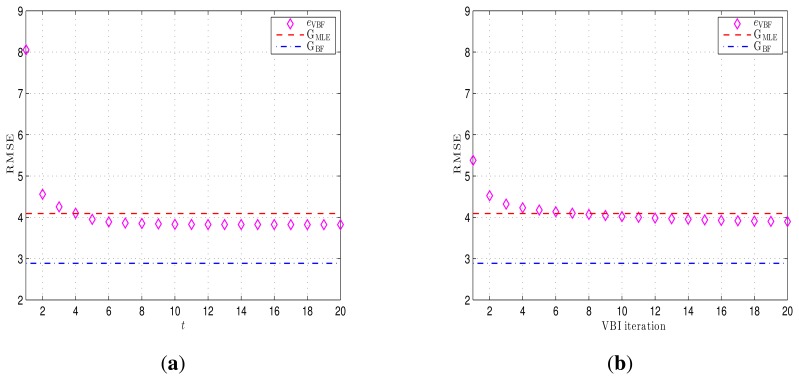
Convergence properties of the VBF scheme. The unit on the *y*-axis is meters. **(a)** Achieved RMSEs of the VBF algorithm at different time instants; **(b)** achieved RMSEs of the VBI algorithm *versus* a different iteration number at one time instant.

The RMSEs achieved by the VBF algorithm *versus* various time instants are shown in Figure 9a, wherein it is assumed that the VBI algorithm converges at each time instant t. As shown in Figure 9a, after some time instants, the VBF scheme can fast track and capture the trajectory of the moving target. At the same time, the final RMSE is lower than the MLE-based CRLB G_MLE_ in the given scenario.

The second case is to examine the convergence of the VBI algorithm for target position estimation, *i.e.*, the RMSEs of position estimation errors achieved by the VBI algorithm versus various variational inference iterations within one time instant of the VBF algorithm. (The VBI algorithm is incorporated into the BF framework to validate a VBF algorithm. See Steps 7–19 in Algorithm 1 for more details.)

Note that, in order to clearly demonstrate the property of the VBI algorithm, in this case, the RMSEs are calculated after the VBF scheme converges (*e.g.*, *t* ≥ 10). As shown in Figure 9b, the VBI-based position estimation error converges quickly to a lower and stable value after a finite number of iterations, which is also lower than G_MLE_.

## 6. Concluding Remarks

In this paper, a MDBN model is first introduced to characterize the target movement problem in WSNs, which incorporates those underlying statistical characteristics in both the target movement and random observations, thus providing a general Bayesian network for the online target tracking. Due to the existence of a nonlinear measurement function in observations, practical online mobile target tracking is difficult by employing the classical BF framework. Hence, a mean-field VBF algorithm is proposed to deduce the approximation for each individual posterior pdf for various system states of the defined MDBN, through optimizing the corresponding KLD.

A uniform solution for each posterior approximation is derived, which is sufficiently formulated with the MB of each system variable. Furthermore, in practical online mobile target tracking applications, since the prior velocity mean is generally unknown in advance, a joint optimization for the prior mean and the hidden velocity variable is therefore incorporated into the VBI iteration to enable online velocity tracking within the online mobile target tracking scheme. Besides, considering the time-varying precision of RSS measurements, a Wishart hyperprior is utilized to characterize this precision's randomness.

On the other hand, the corresponding CRLBs for the VBF-based online mobile target tracking scheme is analyzed. Our analysis reveals that, if we only consider the time-varying measurement precision, the CRLB is just dependent on its expectation. More specifically, the final VBF accuracy scales linearly with this expectation. Moreover, if this expectation is known beforehand, the online mobile target tracking scheme (containing, but is not limited to, the VBF) can achieve an equivalent tracking accuracy with lower computation cost when the time-varying precision is replaced by its expectation. In addition, since the proposed VBF algorithm exploits the potential state information in the online target tracking scheme via the general MDBN model, it can achieve lower RMSE than the MLE-based scheme, as expected. A condition (see Equation (42)) is also provided under which the Bayesian tracking scheme, such as the VBF algorithm, will be degenerated into the MLE-based one. Finally, simulation results are presented to corroborate that the proposed VBF based online mobile target tracking scheme can be utilized to achieve reasonable tracking accuracy from the RSS measurements in the WSNs.

In the future, we are planning to set up an experimental test bed by using Wi-Fi equipment in an indoor environment to verify the proposed VBF-based mobile target tracking from the RSS measurements.

## Figures and Tables

**Table 1.** Simulation Settings.

	**B1**	**B2**	**B3**	**B4**	**B5**
**U**	1/10**I**^†^	1/25**I** : 10**I**	1/10**I**	1/10**I**	1/10**I**
**V**	1/10**I**	1/30**I** : 10**I**	1/10**I**	1/10**I**	1/10**I**
υ [m/s]	0 : 25**I**	10**I**	10**I**	10**I**	10**I**
Σ	1/5**I**	1/5**I**	1/5**I**	1/5**I**	1/5**I**
*β*	10	10	10	10	10
W	1/1500	1/1500	1/2000 : 1/1000	1/1500	1/1500
*ψ*	80	80	60 : 100	80	80
*M_t_*	6	6	6	3 : 15	6

† Here, **I** represents the identity matrix.

**Table 2.** Achieved RMSEs over different movement modes.

	**0.1†**	**5**	**10**	**15**	**25**	**20**
0.1*	1.53, 3.15, 4.07 ∗	2.50, 3.66, 4.09	2.88, 3.82, 4.11	3.11, 3.94, 4.12	3.24, 3.96, 4.11	3.37, 4.06, 4.09
5	2.50, 3.19, 4.10	2.88, 3.79, 4.09	3.09, 3.93, 4.08	3.24, 3.96, 4.10	3.34, 4.04, 3.54	3.43, 4.17, 3.55
10	2.88, 3.40, 4.09	3.09, 3.74, 4.07	3.25, 3.94, 4.11	3.35, 3.95, 4.10	3.44, 4.03, 4.09	3.50, 4.05, 4.08
15	3.10, 3.47, 4.11	3.24, 3.66, 4.08	3.35, 4.01, 4.08	3.43, 4.03, 4.11	3.49, 4.11, 4.10	3.55, 4.12, 4.10
20	3.26, 3.56, 4.09	3.37, 3.84, 4.12	3.46, 4.07, 4.12	3.51, 4.13, 4.08	3.53, 4.14, 4.11	3.60, 4.24, 4.07
25	3.85, 3.64, 4.10	3.46, 3.92, 4.09	3.49, 4.09, 4.07	3.54, 4.12, 4.12	3.60, 4.13, 4.12	3.64, 4.17, 4.13
30	3.44, 3.73, 4.08	3.51, 3.92, 4.11	3.58, 4.17, 4.10	3.60, 4.20, 4.12	3.61, 4.21, 4.12	3.64, 4.26, 4.13

† These raw values denote various $\sigma_{\text{u}}^{2}$, wherein we assume $\text{U}=\sigma_{\text{u}}^{-2}\text{I}$;

‡ these column values denote various $\sigma_{\text{v}}^{2}$, wherein we assume $\text{V}=\sigma_{\text{v}}^{-2}\text{I}$;

* this group of three values denotes ${\text{G}}_{\text{BF}}^{\text{x}}$, *e*_VBF_, ${\text{G}}_{\text{MLE}}^{\text{x}}$, respectively. The associated unit is meters.

**Table 3.** Achieved RMSEs over different levels of shadow fading.

	**60 ^†^**	**70**	**80**	**90**	**100**
0.5 ∗ 10^-3 ‡^	3.28, 4.57, 5.45*	3.16, 4.37, 5.04	3.06, 4.17, 4.70	2.99, 3.96, 4.50	2.90, 3.78, 4.21
0.6 ∗ 10^-3^	3.15, 4.30, 4.98	3.03, 4.13, 4.63	2.93, 3.78, 4.31	2.84, 3.60, 4.07	2.75, 3.40, 3.84
0.7 ∗ 10^-3^	3.02, 3.96, 4.59	2.91, 3.77, 4.28	2.81, 3.50, 3.99	2.72, 3.37, 3.75	2.63, 3.56, 3.21
0.8 ∗ 10^-3^	2.92, 3.84, 4.28	2.81, 3.51, 4.00	2.71, 3.33, 3.74	2.61, 3.14, 3.51	2.52, 3.00, 3.32
0.9 ∗ 10^-3^	2.83, 3.64, 4.05	2.72, 3.41, 3.76	2.62, 3.18, 3.54	2.52, 3.01, 3.32	2.43, 2.83, 3.14
1.0 ∗ 10^-3^	2.75, 3.43, 3.85	2.63, 3.26, 3.56	2.53, 2.97, 3.34	2.43, 2.81, 3.14	2.35, 2.67, 2.98

† These raw numbers denote various values of the DOF *ψ*;

‡ These column numbers denote various values of the scale W;

* This group of three values denotes G_BF_, *e*_VBF_ and G_MLE_, respectively. Note that, the associated unit is meters.

## Appendix

### A. General Approximation Formulation

Following Equation (10), a more concise expression for the general approximation distribution is given by:

$$\begin{array}{ll} q\left(\alpha_{t}^{i}\right) & \propto \exp{\langle \ln p\left(z_{1:t},\alpha_{t}\right)\rangle }_{\prod_{j\neq i}q\left(\alpha_{t}^{j}\right)}\propto \exp{\langle \ln\;\left(p\left({\text{z}}_{t}|\alpha_{t}\right)p\left(\alpha_{t}|z_{1:t-1}\right)\right)\rangle }_{\prod_{j\neq i}q\left(\alpha_{t}^{j}\right)}\\ & \propto \exp{\langle \ln\;\left(p\left(z_{t}|\alpha_{t}\right)\int p\left(\alpha_{t}|\alpha_{t-1}\right)p\left(\alpha_{t-1}|z_{1:t-1}\right)\text{d}\alpha_{t-1}\right)\rangle }_{\prod_{j\neq i}q\left(\alpha_{t}^{j}\right)}\\ & \propto \exp{\langle \ln\;\left(p\left(z_{t}|\alpha_{t}\right)p\left({\tilde{\alpha }}_{t}^{n}\right)\int p\left(\alpha_{t}^{n}|\alpha_{t-1}^{n}\right)p\left(\alpha_{t-1}^{n}|z_{1:t-1}\right)\text{d}\alpha_{t-1}^{n}\right)\rangle }_{\prod_{j\neq i}q\left(\alpha_{t}^{j}\right)} \end{array}$$

Here, the variable $\alpha_{t}^{n}$ denotes the Markov variable as defined in *Definition* 2, for example *μ*_t_, while ${\tilde{\alpha }}_{t}^{n}≔\alpha_{t}\backslash \alpha_{t}^{n}$ is defined as its complementary variables, which are independent of its previous state ${\tilde{\alpha }}_{t}^{n}$.

$$\begin{array}{ll} & \approx \exp{\langle \ln\;\left(p\left(z_{t}|\alpha_{t}\right)p\left({\tilde{\alpha }}_{t}^{n}\right)\int p\left(\alpha_{t}^{n}|\alpha_{t-1}^{n}\right)q\left(\alpha_{t-1}^{n}\right)\text{d}\alpha_{t-1}^{n}\right)\rangle }_{\prod_{j\neq i}q\left(\alpha_{t}^{j}\right)}\\ & =\exp{\langle \ln\;\left(p\left(z_{t}|\alpha_{t}\right)p\left({\tilde{\alpha }}_{t}^{n}\right)q_{p}\left(\alpha_{t}^{n}\right)\right)\rangle }_{\prod_{j\neq i}q\left(\alpha_{t}^{j}\right)} \end{array}$$

Here, *q_p_* $\left(\alpha_{t}^{n}\right)$ is defined as its approximate prediction pdf.

$$\begin{array}{cc} & =\exp{\langle \ln\;\left(p\left(\text{B}\left(\alpha_{t}^{i}\right)|\alpha_{t}^{i}\right)p\left(\alpha_{t}^{i}\right)\prod_{j\notin I\left(\alpha_{t}^{i}\right),j\neq i}p\left(\alpha_{t}^{j}\right)\right)\rangle }_{\prod_{j\neq i}q\left(\alpha_{t}^{j}\right)} \end{array}$$

Here, $\text{I}\left(\alpha_{t}^{i}\right):=\left\{j|\alpha_{t}^{j}\in \text{B}\left(\alpha_{t}^{i}\right)\right\}$ is defined as the index set of the MB $\text{B}\left(\alpha_{t}^{i}\right)$.

(A1.1) $$\begin{array}{cc} & =\underset{⫫q\left(\alpha_{t}^{j}\right)}{\underset{︸}{\exp{\langle \ln\;\left(\prod_{j\notin \text{I}\left(\alpha_{t}^{i}\right),j\neq i}p\left(\alpha_{t}^{j}\right)\right)\rangle }_{\prod_{j\neq i}q\left(\alpha_{t}^{j}\right)}}}\cdot \exp{\langle \ln\;\left(p\left(\text{B}\left(\alpha_{t}^{i}\right)|\alpha_{t}^{i}\right)p\left(\alpha_{t}^{i}\right)\right)\rangle }_{\prod_{j\in \text{I}\left(\alpha_{t}^{i}\right)}q\left(\alpha_{t}^{j}\right)}\\ & \propto \exp{\langle \ln\;\left(p\left(\text{B}\left(\alpha_{t}^{i}\right)|\alpha_{t}^{i}\right)p\left(\alpha_{t}^{i}\right)\right)\rangle }_{q\left(\text{B}\left(\alpha_{t}^{i}\right)\right)}=\exp{\langle \ln p\left(\alpha_{t}^{i},\text{B}\left(\alpha_{t}^{i}\right)\right)\rangle }_{q\left(\text{B}\left(\alpha_{t}^{i}\right)\right)}, \end{array}(\text{A}.1)$$

where $\text{I}(\alpha_{t}^{i})≔\{j|\alpha_{t}^{j}\in \text{B}(\alpha_{t}^{i})\}$ is defined as the index set of the MB $\text{B}(\alpha_{t}^{i})$

### B. Specific Variational Approximation

Based on Equation (12), the approximation to the posterior pdf *q*(x_*t*_) can be deduced as:

$$\begin{array}{l} q\left(x_{t}\right)\propto \exp{\langle \ln p\left(x_{t},B\left(x_{t}\right)\right)\rangle }_{q\left(B\left(x_{t}\right)\right)}=\exp{\langle \ln p\left(x_{t},z_{t},\mu_{t},\Lambda_{t},{\text{w}}_{t}\right)\rangle }_{q\left(z_{t},\mu_{t},\Lambda_{t},{\text{w}}_{t}\right)}\\ =\underset{⫫q\left(x_{t}\right)}{\underset{︸}{\exp\left({\langle \ln\;\left(q_{p}^{\upsilon }\left(\mu_{t}\right)p\left(\Lambda_{t}\right)\right)\rangle }_{z_{t},\mu_{t},\Lambda_{t}}\right)}}\cdot \exp\left({\langle \ln\;\left(p\left(z_{t}|x_{t},w_{t}\right)p\left(x_{t}|\mu_{t},\Lambda_{t}\right)\right)\rangle }_{\mu_{t},\Lambda_{t},{\text{w}}_{t}}\right)\\ \propto \exp\left({\langle \ln p\left(x_{t}|\mu_{t},\Lambda_{t}\right)\rangle }_{\mu_{t},\Lambda_{t}}\right)\cdot \exp\left({\langle \ln p\left(z_{t}|x_{t},w_{t}\right)\rangle }_{{\text{w}}_{t}}\right)\\ =\exp\left({\langle \ln p\left(x_{t}|\mu_{t},\Lambda_{t}\right)\rangle }_{\mu_{t},\Lambda_{t}}\right)\cdot \underset{\text{Assume}\;{\text{z}}_{t}^{i}⫫\;{\text{z}}_{t}^{j},\;\text{if}\;\left(i,j\right)\;\in \;{𝒮}_{t}\;\text{and}\;i\neq j.}{\underset{︸}{\prod_{i=1}^{M_{t}}\exp\left({\langle \ln\text{N}\left(z_{t}^{i}|h^{i}\left(x_{t}\right),{\text{w}}_{t}\right)\rangle }_{{\text{w}}_{t}}\right)}}\\ \propto \exp\left(-\frac{1}{2}{\langle {\left(x_{t}-\mu_{t}\right)}^{⊤}\Lambda_{t}\left(x_{t}-\mu_{t}\right)\rangle }_{\mu_{t},\Lambda_{t}}\right)\cdot \prod_{i=1}^{M_{t}}\exp\left(-\frac{1}{2}{\langle {\text{w}}_{t}\rangle }_{{\text{w}}_{t}}{\left({\text{z}}_{t}^{i}-h^{i}\left(x_{t}\right)\right)}^{2}\right)\\ =\exp\left(-\frac{1}{2}\text{tr}\left({\langle \Lambda_{t}\rangle }_{\Lambda_{t}}{\langle \left(x_{t}-\mu_{t}\right){\left(x_{t}-\mu_{t}\right)}^{⊤}\rangle }_{\mu_{t}}\right)\right)\cdot \prod_{i=1}^{M_{t}}\text{N}\left(z_{t}^{i}|h^{i}\left(x_{t}\right),\langle {\text{w}}_{t}\rangle \right) \end{array}$$

$\langle \mu_{t},\mu_{t}^{Τ}\rangle =\langle \mu_{t}\rangle \langle \mu_{t}^{Τ}\rangle +\mathrm{cov}(\mu_{t})$ and cov(*μ_t_*) can be read as a constant:

(B.1) $$\propto \text{N}\left(x_{t}|\langle \mu_{t}\rangle ,\langle \Lambda_{t}\rangle \right)\cdot \prod_{i=1}^{M_{t}}\text{N}\left(z_{t}^{i}|h^{i}\left(x_{t}\right),\langle {\text{w}}_{t}\rangle \right).\;$$

Based on Equation (12), the approximation to the posterior pdf *q*(***μ****_t_*) can be deduced as:

$$\begin{array}{l} q\left(\mu_{t}\right)\propto \exp{\langle \ln p\left(\mu_{t},B\left(\mu_{t}\right)\right)\rangle }_{q\left(B\left(\mu_{t}\right)\right)}=\exp{\langle \ln p\left(\mu_{t},x_{t},\Lambda_{t},\upsilon_{t}\right)\rangle }_{q\left(x_{t},\Lambda_{t},\upsilon_{t}\right)}\\ =\underset{⫫q\left(\mu_{t}\right)}{\underset{︸}{\exp\left({\langle \ln\left(p\left(\upsilon_{t}\right)p\left(\Lambda_{t}\right)\right)\rangle }_{\upsilon_{t},\Lambda_{t}}\right)}}\cdot \exp\left({\langle \ln\left(p\left(x_{t}|\mu_{t},\Lambda_{t}\right)q_{p}\left(\mu_{t}|\upsilon_{t},U\right)\right)\rangle }_{x_{t},\Lambda_{t},\upsilon_{t}}\right)\\ \propto \exp\left({\langle \ln q_{p}\left(\mu_{t}|\upsilon_{t},U\right)\rangle }_{\upsilon_{t}}\right)\cdot \exp\left({\langle \ln p\left(x_{t}|\mu_{t},\Lambda_{t}\right)\rangle }_{x_{t},\Lambda_{t}}\right)\\ \propto \exp\left(-\frac{1}{2}{\langle {\left(\mu_{t}-\mu_{t}^{p}\right)}^{⊤}U_{t}^{p}\left(\mu_{t}-\mu_{t}^{p}\right)\rangle }_{\upsilon_{t}}\right)\cdot \exp\left(-\frac{1}{2}{\langle {\left(x_{t}-\mu_{t}\right)}^{⊤}\Lambda_{t}\left(x_{t}-\mu_{t}\right)\rangle }_{x_{t},\Lambda_{t}}\right)\\ \propto \exp\left(-\frac{1}{2}{\left(\mu_{t}-{\langle \mu_{t}^{p}\rangle }_{\upsilon_{t}}\right)}^{⊤}U_{t}^{p}\left(\mu_{t}-{\langle \mu_{t}^{p}\rangle }_{\upsilon_{t}}\right)\right)\cdot \exp\left(-\frac{1}{2}\text{tr}\left({\langle \Lambda_{t}\rangle }_{\Lambda_{t}}{\langle \left(x_{t}-\mu_{t}\right){\left(x_{t}-\mu_{t}\right)}^{⊤}\rangle }_{x_{t}}\right)\right)\\ \propto \text{N}\left(\mu_{t}|{\langle \mu_{t}^{p}\rangle }_{\upsilon_{t}},U_{t}^{p}\right)\cdot \text{N}{\left(\mu_{t}|\langle x_{t}\rangle ,\langle \Lambda_{t}\rangle \right)|}_{{\langle \mu_{t}^{p}\rangle }_{\upsilon_{t}}≔\mu_{t-1}^{*}+\langle \upsilon_{t}\rangle } \end{array}$$

*Lemma* 1: The product of two Gaussian pdfs is also a Gaussian pdf: The associated expectation and precision can be deduced by calculating the first order and second order derivatives of the logarithm of the Gaussian pdf; respectively:

(B.2) $$≔\text{N}{\left(\mu_{t}|\mu_{t}^{*},U_{t}^{*}\right)|}_{\mu_{t}^{*}={\left(U_{t}^{*}\right)}^{-1}\left(U_{t}^{p}{\langle \mu_{t}^{p}\rangle }_{\upsilon_{t}}+\langle \Lambda_{t}\rangle \langle x_{t}\rangle \right),U_{t}^{*}=U_{t}^{p}+\langle \Lambda_{t}\rangle }.$$

Based on Equation (12), the approximation to the posterior pdf *q*(**μ**_t_) can be deduced as:

(B.3) $$\begin{array}{l} q\left(\Lambda_{t}\right)\propto \exp{\langle \ln p\left(\Lambda_{t},B\left(\Lambda_{t}\right)\right)\rangle }_{q\left(B\left(\Lambda_{t}\right)\right)}=\exp{\langle \ln p\left(\Lambda_{t},x_{t},\mu_{t}\right)\rangle }_{q\left(x_{t},\mu_{t}\right)}\\ =\underset{⫫q\left(\Lambda_{t}\right)}{\underset{︸}{\exp\left({\langle \ln q_{p}^{\upsilon }\left(\mu_{t}\right)\rangle }_{\mu_{t}}\right)}}\cdot \exp\left({\langle \ln\left(p\left(x_{t}|\mu_{t},\Lambda_{t}\right)p\left(\Lambda_{t}|\sum ,\beta \right)\right)\rangle }_{x_{t},\mu_{t}}\right)\\ \propto 𝒲\left(\Lambda_{t}|\sum ,\beta \right)\cdot \exp\left({\langle \ln p\left(x_{t}|\mu_{t},\Lambda_{t}\right)\rangle }_{x_{t},\mu_{t}}\right)\\ \propto {\left|\Lambda_{t}\right|}^{\frac{\beta -{\text{D}}_{x}-1}{2}}\exp\left(-\frac{1}{2}\text{tr}\left(\sum^{-1}\Lambda_{t}\right)\right)\cdot {\left|\Lambda_{t}\right|}^{1/2}\exp\left(-\frac{1}{2}\text{tr}\left(\Lambda_{t}{\langle \left(x_{t}-\mu_{t}\right){\left(x_{t}-\mu_{t}\right)}^{⊤}\rangle }_{x_{t},\mu_{t}}\right)\right)\\ ={\left|\Lambda_{t}\right|}^{\frac{\beta +1-{\text{D}}_{x}-1}{2}}\exp{\left(-\frac{1}{2}\text{tr}\left(\left(\sum^{-1}+x_{t}\right)\Lambda_{t}\right)\right)|}_{x_{t}≔{\langle \left(x_{t}-\mu_{t}\right){\left(x_{t}-\mu_{t}\right)}^{⊤}\rangle }_{x_{t,\mu_{t}}}}\\ ={\left|\Lambda_{t}\right|}^{\frac{\beta *-{\text{D}}_{x}-1}{2}}\exp{\left(-\frac{1}{2}\text{tr}\left({\left(\sum_{t}^{*}\right)}^{-1}\Lambda_{t}\right)\right)|}_{\beta *≔\beta +1,\sum_{t}^{*}≔{\left(\sum^{-1}+x_{t}\right)}^{-1}}\\ \propto 𝒲\left(\Lambda_{t}|\sum_{t}^{*},\beta *\right). \end{array}$$

Based on Equation (12), the approximation to the posterior pdf *q*(*v_t_*) can be deduced as:

(B.4) $$\begin{array}{l} q\left(\upsilon_{t}\right)\propto \exp{\langle \ln p\left(\upsilon_{t},\text{B}\left(\upsilon_{t}\right)\right)\rangle }_{q\left(\text{B}\left(\upsilon_{t}\right)\right)}=\exp{\langle \ln p\left(\upsilon_{t},\mu_{t}\right)\rangle }_{q\left(\mu_{t}\right)}\\ =\exp\left({\langle \ln\left(q_{p}\left(\mu_{t}|\upsilon_{t},\text{U}\right)p\left(\upsilon_{t}|\upsilon ,\text{V}\right)\right)\rangle }_{\mu_{t}}\right)=\text{N}\left(\upsilon_{t}|\upsilon ,\text{V}\right)\cdot \exp\left({\langle \ln q_{p}\left(\mu_{t}|\upsilon_{t},\text{U}\right)\rangle }_{\mu_{t}}\right)\\ \propto \text{N}\left(\upsilon_{t}|\upsilon ,\text{V}\right)\cdot \exp\left(-\frac{1}{2}{\langle {\left(\mu_{t}-\mu_{t-1}^{*}-\upsilon_{t}\right)}^{Τ}{\text{U}}_{t}^{p}\left(\mu_{t}-\mu_{t-1}^{*}-\upsilon_{t}\right)\rangle }_{\mu_{t}}\right)\\ \propto \text{N}\left(\upsilon_{t}|\upsilon ,\text{V}\right)\cdot \exp\left(-\frac{1}{2}{\left({\langle \mu_{t}\rangle }_{\mu_{t}}-\mu_{t-1}^{*}-\upsilon_{t}\right)}^{Τ}{\text{U}}_{t}^{p}\left({\langle \mu_{t}\rangle }_{\mu_{t}}-\mu_{t-1}^{*}-\upsilon_{t}\right)\right)\\ \propto \text{N}\left(\upsilon_{t}|\upsilon ,\text{V}\right)\text{N}\left(\upsilon_{t}|\langle \mu_{t}\rangle -\mu_{t-1}^{*},{\text{U}}_{t}^{p}\right)=\text{N}\left(\upsilon_{t}|\upsilon ,\text{V}\right)\text{N}\left(\upsilon_{t}|{\langle \upsilon_{t}^{\#}\rangle }_{\mu_{t}},{\text{U}}_{t}^{p}\right)|\begin{array}{c} \text{Define}\;\upsilon_{t}^{\#}≔\mu_{t}-\mu_{t-1}^{*}.\\ {\langle \upsilon_{t}^{\#}\rangle }_{\mu_{t}}≔\langle \mu_{t}\rangle -\mu_{t-1}^{*} \end{array}\\ ≔\text{N}\left(\upsilon_{t}|\upsilon_{t}^{*},{\text{V}}_{t}^{*}\right)|\upsilon_{t}^{*}={\left({\text{v}}_{t}^{*}\right)}^{-1}\left({\text{U}}_{t}^{P}{\langle \upsilon_{t}^{\#}\rangle }_{\mu_{t}}+\text{v}\upsilon \right),{\text{v}}_{t}^{*}≔{\text{U}}_{t}^{P}+\text{v}{\left({\left(U_{t-1}^{*}\right)}^{-1}+{\text{U}}^{-1}\right)}^{-1}+{\text{v}}^{\cdot } \end{array}$$

Based on Equation (12), the approximation to the posterior pdf *q*(w*_t_*) can be deduced as:

(B.5) $$\begin{array}{cc} q\left({\text{w}}_{t}\right) & \propto \exp{\langle \ln p\left({\text{w}}_{t},\text{B}\left({\text{w}}_{t}\right)\right)\rangle }_{q\left(\text{B}\left({\text{w}}_{t}\right)\right)}=\exp{\langle \ln p\left({\text{w}}_{t},{\text{z}}_{t},{\text{x}}_{t}\right)\rangle }_{q\left({\text{x}}_{t}\right)}\\ & =\underset{\text{Cons}\tan\text{t}}{\underset{︸}{\exp\left({\langle \ln p\left({\text{x}}_{t}|\mu_{t},\Lambda_{t}\right)\rangle }_{{\text{x}}_{t}}\right)}}\cdot \exp\left({\langle \ln\left(p\left({\text{z}}_{t}|{\text{x}}_{t},{\text{w}}_{t}\right)p\left({\text{w}}_{t}|\text{W},\psi \right)\right)\rangle }_{{\text{x}}_{t}}\right)\\ & \propto W\left({\text{w}}_{t}|\text{W},\psi \right)\cdot \underset{\text{Assume}\;{\text{z}}_{t}^{i}⫫{\text{z}}_{t}^{j},\forall i\neq j}{\underset{︸}{\exp\left({\langle \ln\text{N}\left({\text{z}}_{t}|{\text{x}}_{t},{\text{w}}_{t}\right)\rangle }_{{\text{x}}_{t}}\right)}}\\ & \propto W\left({\text{w}}_{t}|\text{W},\psi \right)\cdot \prod_{i=1}^{M_{t}}{\left|{\text{w}}_{t}\right|}^{1/2}\exp\left(-\frac{1}{2}{\langle {\text{w}}_{t}{\left({\text{z}}_{t}^{i}-h^{i}\left({\text{x}}_{t}\right)\right)}^{2}\rangle }_{{\text{x}}_{t}}\right)\\ & =W\left({\text{w}}_{t}|\text{W},\psi \right)\cdot \prod_{i=1}^{M_{t}}{\left|{\text{w}}_{t}\right|}^{1/2}\exp\left(-\frac{1}{2}{\left({\text{W}}_{t}^{i}\right)}^{-1}{\text{w}}_{t}\right)|{\text{w}}_{t}^{i}={\langle {\left({\text{z}}_{t}^{i}-h^{i}\left({\text{x}}_{t}\right)\right)}^{2}\rangle }_{{\text{x}}_{t}}^{-1}\\ & =W\left({\text{w}}_{t}|\text{W},\psi \right)\cdot {\left|{\text{w}}_{t}\right|}^{1/2}\exp\left(-\frac{1}{2}{\text{w}}_{t}\sum_{i=1}^{M_{t}}{\left({\text{W}}_{t}^{i}\right)}^{-1}\right)\\ & \propto W\left({\text{w}}_{t}|\text{W},\psi \right)\cdot W\left({\text{w}}_{t}|{\text{W}}_{t}^{\#},\psi_{t}^{\#}\right)|{\text{W}}_{t}^{\#}={\left(\sum_{i=1}^{M_{t}}{\left(w_{t}^{i}\right)}^{-1}\right)}^{-1},\psi_{t}^{\#}=M_{t}+2\\ & ≔W\left({\text{w}}_{t}|{\text{W}}_{t}^{*},\psi_{t}^{*}\right)|\psi_{t}^{*}=\psi +\psi_{t}^{*}-2=\psi +M_{t},{\text{w}}_{t}^{*}={\left({\left({\text{w}}_{t}^{\#}\right)}^{-1}+{\text{w}}^{-1}\right)}^{-1}{\left(\sum_{i=1}^{M_{t}}{\left({\text{w}}_{t}^{i}\right)}^{-1}+{\text{w}}^{-1}\right)}^{-1.} \end{array}$$

### C. Derivation of the MLE-Based FIM

Before deriving the MLE-based FIM ${\text{R}}_{\text{MLE},t}^{\text{x}}$, we give a useful formulation at first, *i.e.*, the derivative of the likelihood function as:

(C.1) $$\begin{array}{l} \nabla_{{\text{x}}_{t}}\ln p\left({\text{z}}_{t}^{s}|{\text{x}}_{t},{\text{w}}_{t}\right)=-\frac{1}{2}{\text{w}}_{t}\cdot \nabla_{{\text{x}}_{t}}\left(\sum_{i=1}^{M_{t}}{\left({\text{z}}_{t}^{i}-h^{i}\left({\text{x}}_{t}\right)\right)}^{2}\right)\\ =-{\text{w}}_{t}\cdot \sum_{i=1}^{M}(({\text{z}}_{t}^{i}-h^{i}({\text{x}}_{t}))\underset{{\text{g}}_{t}^{i}}{\underset{︸}{\nabla_{{\text{x}}_{t}}\left({\text{z}}_{t}^{i}-h^{i}\left({\text{x}}_{t}\right)\right)}}), \end{array}$$

where the associated derivative $g_{t}^{i}$ is further deduced as:

(C.2) $$\begin{array}{c} {\text{g}}_{t}^{i}=\nabla_{{\text{x}}_{t}}\left({\text{z}}_{t}^{i}-\phi +10\gamma \log_{10}{\left\|{\text{s}}_{t}^{i}-{\text{x}}_{t}\right\|}_{2}\right)\\ =\frac{10\gamma }{\ln10}{\left\|{\text{s}}_{t}^{i}-{\text{x}}_{t}\right\|}_{2}^{-2}\left({\text{x}}_{t}-{\text{s}}_{t}^{i}\right). \end{array}$$

Thus, the MLE-based FIM ${\text{R}}_{\text{MLE},t}^{\text{x}}$ can be formulated as (here, we turn to using $\text{J}=\text{E}\left\{\nabla_{\text{x}t}(•)\cdot \nabla_{{\text{x}}_{t}^{⊤}}(•)\right\}$ as the FIM formulation):

(C.3) $$\begin{array}{l} {\text{R}}_{\text{MLE},t}^{\text{x}}=-\sum_{i=1}^{M_{t}}{\text{E}}_{{\text{z}}_{t},\alpha_{t}}\left\{\nabla_{{\text{x}}_{t},{\text{x}}_{t}^{⊤}}\ln p\left({\text{z}}_{t}^{i}|{\text{x}}_{t},{\text{w}}_{t}\right)\right\}\\ =\sum_{i=1}^{M_{t}}{\text{E}}_{{\text{z}}_{t},\alpha_{t}}\left\{\nabla_{{\text{x}}_{t}}\ln p\left({\text{z}}_{t}^{i}|{\text{x}}_{t},{\text{w}}_{t}\right)\cdot \nabla_{{\text{x}}_{t}^{⊥}}\ln p\left({\text{z}}_{t}^{i}|{\text{x}}_{t},{\text{w}}_{t}\right)\right\}\\ =\sum_{i=1}^{M_{t}}{\text{E}}_{\alpha_{t}}\{{\text{w}}_{t}^{2}\underset{{\text{w}}_{t}^{-1}}{\underset{︸}{{\text{E}}_{{\text{z}}_{t}}\left\{{\left({\text{z}}_{t}^{i}-h^{i}\left({\text{x}}_{t}\right)\right)}^{2}\right\}}}{\text{g}}_{t}^{i}{\text{g}}_{t}^{iΤ}\}\\ ={\text{E}}_{{\text{w}}_{t}}\left\{{\text{w}}_{t}\right\}\sum_{i=1}^{M_{t}}{\text{E}}_{{\text{x}}_{t}}\left\{{\text{g}}_{t}^{i}{\text{g}}_{t}^{iΤ}\right\}\\ ={\left(\frac{10\gamma }{\ln10}\right)}^{2}\text{W}\psi \underset{{\text{A}}_{t}}{\underset{︸}{\sum_{i=1}^{M_{t}}{\text{E}}_{x_{t}}\left\{\frac{\left({\text{x}}_{t}-{\text{s}}_{t}^{i}\right){\left({\text{x}}_{t}-{\text{s}}_{t}^{i}\right)}^{Τ}}{{\left\|{\text{x}}_{t}-{\text{s}}_{t}^{i}\right\|}_{2}^{4}}\right\}}} \end{array}$$

Note that, for the MLE-based FIM, since there is no prior information about the target location, the associated expectation **E**_x*_t_*_ **{•}** is calculated with the real value of x*_t_*, *i.e.*, ${\text{A}}_{t}=\sum_{i=1}^{M_{t}}\frac{\left({\text{x}}_{t}-{\text{s}}_{t}^{i}\right){\left({\text{x}}_{t}-{\text{s}}_{t}^{i}\right)}^{Τ}}{{\left\|{\text{x}}_{t}-{\text{s}}_{t}^{i}\right\|}_{2}^{4}}$
